# Profiling non-coding RNA levels with clinical classifiers in pediatric Crohn’s disease

**DOI:** 10.1186/s12920-021-01041-7

**Published:** 2021-07-29

**Authors:** Ranjit Pelia, Suresh Venkateswaran, Jason D. Matthews, Yael Haberman, David J. Cutler, Jeffrey S. Hyams, Lee A. Denson, Subra Kugathasan

**Affiliations:** 1 Division of Pediatric Gastroenterology, Department of Pediatrics, Emory University School of Medicine and Children’s Healthcare of Atlanta, 1760 Haygood Drive, W-427, Atlanta, GA 30322 USA; 2 Cincinnati Children’s Hospital Medical Center, University of Cincinnati College of Medicine, Cincinnati, OH USA; 3 Sheba Medical Center, Tel-HaShomer, Affiliated With the Tel-Aviv University, Tel-Aviv, Israel; 4 Department of Human Genetics, Emory University, Atlanta, GA USA; 5 Connecticut Children’s Medical Center, Hartford, CT USA

**Keywords:** Differentially expressed non-coding RNAs (DE ncRNAs), Inflammatory bowel disease (IBD), Crohn’s disease (CD)

## Abstract

**Background:**

Crohn’s disease (CD) is a heritable chronic inflammatory disorder. Non-coding RNAs (ncRNAs) play an important role in epigenetic regulation by affecting gene expression, but can also directly affect protein function, thus having a substantial impact on biological processes. We investigated whether non-coding RNAs (ncRNA) at diagnosis are dysregulated during CD at different CD locations and future disease behaviors to determine if ncRNA signatures can serve as an index to outcomes.

**Methods:**

Using subjects belonging to the RISK cohort, we analyzed ncRNA from the ileal biopsies of 345 CD and 71 non-IBD controls, and ncRNA from rectal biopsies of 329 CD and 61 non-IBD controls. Sequence alignment was done (STAR package) using Human Genome version 38 (hg38) as reference panel. The differential expression (DE) analysis was performed with EdgeR package and DE ncRNAs were identified with a threshold of fold change (FC) > 2 and FDR < 0.05 after multiple test corrections.

**Results:**

In total, we identified 130 CD specific DE ncRNAs (89 in ileum and 41 in rectum) when compared to non-IBD controls. Similarly, 35 DE ncRNAs were identified between B1 and B2 in ileum, whereas no differences among CD disease behaviors were noticed in rectum. We also found inflammation specific ncRNAs between inflamed and non-inflamed groups in ileal biopsies. Overall, we observed that expression of *mir1244-2*, *mir1244-3*, *mir1244-*4, and *RN7SL2* were increased during CD, regardless of disease behavior, location, or inflammatory status. Lastly, we tested ncRNA expression at baseline as potential tool to predict the disease status, disease behaviors and disease inflammation at 3-year follow up.

**Conclusions:**

We have identified ncRNAs that are specific to disease location, disease behavior, and disease inflammation in CD. Both ileal and rectal specific ncRNA are changing over the course of CD, specifically during the disease progression in the intestinal mucosa. Collectively, our findings show changes in ncRNA during CD and may have a clinical utility in early identification and characterization of disease progression.

**Supplementary Information:**

The online version contains supplementary material available at 10.1186/s12920-021-01041-7.

## Background

Inflammatory bowel disease (IBD) is a disorder affecting the intestines with two prominent disease types; ulcerative colitis (UC) and Crohn’s disease (CD), whereby UC is confined mostly to the colonic mucosa with persistent chronic inflammation [1] and CD is a transmural disease affecting the entire gastrointestinal tract [2]. Rising incidence of IBD have been attributed to the gut-microbiome interactions, genetic predispositions, and environmental triggers [3], but more recently attention has been placed on epigenetic mechanisms [4]. Non-coding RNAs (ncRNAs) play an important role in epigenetic regulation by affecting gene expression but can also directly affect protein function, thus having a substantial impact on biological processes [5]. Though there are many types of ncRNAs, the defining characteristic of a ncRNA is their length; ranging from ~ 22 nucleotides (nts) to > 200nts [6], and for micro-RNAs, 20–25 nts [7].

The Montreal classification system defines the CD behaviors as B1, a non-stricturing/non-penetrating disease; B2 as stricturing; and B3 as penetrating disease. Similarly, the site of CD manifestation is also stratified into three main locations, L1 (ileal), L2 (colonic), and L3 (ileocolonic) [8]. Previous CD studies suggest that environmental factors can modulate pre-disposition and affect disease outcomes [9], likely through genetic and/or epigenetic mechanisms. However, little is known about the role epigenetics or ncRNA expression might play in influencing specific disease behavior, location, or inflammatory status during CD.

In fact, very few studies have investigated the role of ncRNA on epigenetic mechanisms to determine whether they are playing a beneficial or detrimental role in IBD development and progression. Notably, earlier we performed the first large scale CD ncRNA analysis in IBD and identified two differentially expressed (DE) up-regulated lncRNAs, *LINC01272* and *HNF4A-AS1* in CD patients [10]. Likewise, a few other studies have analyzed smaller datasets and observed changes in IBD specific ncRNAs, i.e. LINC01272 [10], DIO3OS [11], and KIF9-AS [11] using RT-PCR in intestinal tissues and plasma samples in IBD. In particular, the expression of these ncRNAs was greater in IBD patients when compared to controls [11]. However, none of these studies explored whether the ncRNAs expression are specific to tissue types, CD disease behaviors, disease location or disease inflammation. Therefore, in this study our goal was to identify the ncRNAs associated with tissue location (ileum vs. rectum), CD disease behaviors (among B1, B2 and B3), and inflammation on disease location (among L1, L2 and L3) during CD by utilizing CD participants from the largest RISK cohort clinical and disease characteristics.

Using the RISK cohort [12], we evaluated high-density ncRNA transcriptomic profiles from 735 samples (345 are from ileal and 390 are from rectal biopsies) with well-defined 5-year patient follow-up and clinical metadata. We assessed whether CD specific ncRNA expressions were consistent across tissue location (ileum vs. rectum) and investigated whether changes in ncRNA levels are representative of CD disease behaviors (B1, B2 and B3). Lastly, we tested whether changes in ncRNA levels have the potential to distinguish inflammatory status from disease location within CD patients. Our results show a dysregulation in ncRNA abundance in different mucosal location during distinct stages of CD progression (thus the disease behavior) that are potentially useful in future clinical companion diagnosis.

## Methods

### Cohort

All samples used in this study were part of the RISK study (Risk Stratification and Identification of Immunogenetic and Microbial Markers of Rapid Disease Progression in Children with Crohn’s Disease) [12]. Prior to diagnosis and treatment, confirmation of disease status and extent was histologically evaluated by a physician(s). Ileal and rectum bulk-biopsies were obtained from newly diagnosed CD patients by colonoscopy. Patients with no bowel pathology, negative gut inflammation, and asymptomatic for IBD, were considered as non-IBD controls. Ileal biopsies were obtained from 71 non-IBD controls and 274 CD patients at diagnosis. Similarly, rectal biopsies were obtained from 61 controls and 329 CD patients at diagnosis from the same RISK cohort. Individuals were clinically assigned according to the Montreal classification system, initially at baseline, for CD patients. Physician assessed Montreal classifiers were denoted by disease status such as B1 (inflammatory), B2 (stricturing), or B3 (penetrating); and inflammation location such as L1 (ileal), L2 (colonic), and L3 (ileal-colonic) [13]. Disease severity classifications, demographics, and clinical information were collected for each patient at time of enrollment and during follow-up, are provided in Table 1 along with other patient metrics included age, sex, disease type, disease behavior, inflammatory status and inflammation on disease location.

**Table 1:** Patient clinical characteristics

	Ileum (n = 345)	Rectum (n = 390)	Total (n = 735)
Sex			
Female	130 (37.7%)	151 (38.7%)	281 (38.2%)
Male	215 (62.3%)	239 (61.3%)	454 (61.8%)
Age			
Mean (SD)	12.309 (3.000)	12.249 (3.071)	12.277 (3.036)
Range	1.750—18.000	2.250—17.583	1.750—18.000
Disease type			
CD	274 (79.4%)	329 (84.4%)	603 (82.0%)
Control	71 (20.6%)	61 (15.6%)	132 (18.0%)
Disease behaviors			
B1	233 (67.5%)	262 (67.2%)	495 (67.3%)
B2	27 (7.8%)	44 (11.3%)	71 (9.7%)
B3	14 (4.1%)	23 (5.9%)	37 (5.0%)
Inflammatory status			
Inflamed	254 (73.6%)	166 (42.6%)	420 (57.1%)
Non inflamed	20 (5.8%)	163 (41.8%)	183 (24.9%)
Inflammation location			
L1	51 (14.8%)	2 (0.5%)	53 (7.2%)
L2	56 (16.2%)	43 (11.0%)	99 (13.5%)
L3	147 (42.6%)	121 (31.0%)	268 (36.5%)
Non inflamed	20 (5.8%)	163 (41.8%)	183 (24.9%)

Patient Clinical Characteristics: Metadata of patients' RNA-sequenced comprised of characteristics measured at time of enrollment into study. Measured variables included sex, age, disease type, disease behavior, and inflammatory status

### RNA-sequencing

All biopsies were extracted and processed as previously described [14, 15] with NEBNext Ultra RNA Library Prep Kit used for Illumina RNA sequencing library preparations by following set manufacturer’s recommendations (NEB, Ipswich, MA, USA). Approximately 77% of the 274 CD ileal biopsies, n = 212, were used for our previous publication [10]. These were re-sequenced for a deeper RNAseq, alongside the other ileal samples. Libraries were sequenced on the HiSeq system using Paired End (PE) 150 base pair chemistry by GEMEWIZ, South Plainfield NJ. Whole biopsy RNA sequencing for both ileal and rectal samples was done in a single batch. Read quantification was conducted and aligned to the GENCODE v28 (HG38) reference genome using STAR package [16]. EdgeR was used to analyze Differential Expressed long ncRNAs (DE lncRNAs) [17, 18]. In total, 20,779 ncRNAs were analyzed in both the ileum and rectum datasets. Overall, a list of fifteen types of non-coding RNAs were assessed, where the categorization was based upon their length of nucleotides (Additional file 1: Table S1).

### Study design

The workflow and the overall layout of this study are provided in Additional file 2: Fig. S1, where the total number of samples included and number of differentially expressed ncRNA (DEncRNA) are identified in each comparison. Overall, we examined the differential expression of ncRNA across tissue types, CD disease behaviors, inflammation on disease location obtained from a subset of pediatric CD patient’s intestinal biopsies from RISK study (Additional file 3: Table S2).

### Statistical analysis

Genome-wide differential expression analysis was conducted using computational algorithms with packages EdgeR [17] and SARtools [19] in R Studio version 1.2 [20]. In this study, the DE both up- and down- regulated ncRNAs were defined with FC > 2 and FDR < 0.05 after multiple test corrections and Principal Component Analysis (PCA) were performed using princomp package [21].

### lncRNA-miRNA modeling

Thermodynamic calculations, binding affinities, and base-pair modeling were conducted using IntaRNA version 2.3.0, in conjunction with Vienna RNA package 2.4.9 [2, 22, 23]. Utilizing this server, possible interactions between target mRNAs such as *mir-1244-2*, *mir-1244-3*, *mir-1244-4* and query *RN7SL2 ncRNA* were computed with parameters for the sliding window size set with 150, maximum length of unpaired region with 150, maximum distance of two paired bases with 100, the weight for ED values of target RNA and query RNA were set at 1, and the temperature was set at 37 degrees Celsius. The Heuristic for hybridization end used was also incorporated within the molecular base-pairing predictions.

### Random forest prediction

The total number of samples was split into a training and a validation set. The training sets contained equal number of samples for each comparison. These were randomly selected based on alignment quality, sorting best to worst. Of the selected samples, the training set consisted of 50% of the data whereas the validation sets contained 50% of the remaining data. Using RandomForest, classifiers were built on the training set using n = 100,000 trees, m_try_ set to 2, and disease status, behavior, or inflammation were evaluated. To test each classifier model, five-fold cross-validation was implemented. Each time, samples were arbitrarily selected to train and test the model. Cross-validation folds were fixed, across all comparisons. Accuracies were calculated using confusion matrices of test set class labels and test set predictions. These accuracies demonstrate the overall robustness of each model.

## Results

### ncRNA profiles within mucosal biopsies separate CD from controls

**Fig. 1:**
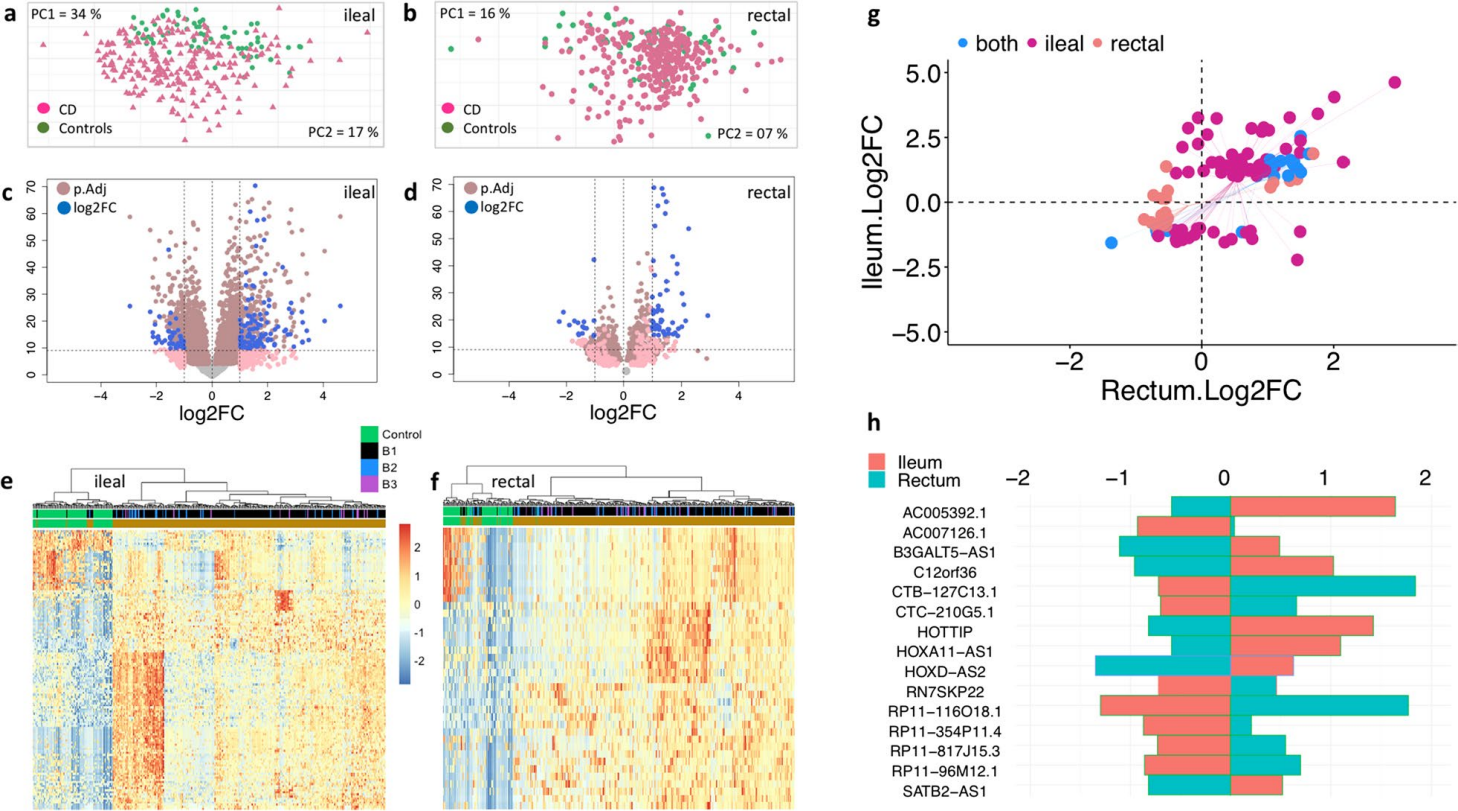
Crohn’s disease versus controls DE ncRNAs. Using princomp, principal components of entire ncRNAs (n = 20,779) were calculated in ileal (n = 345) **a** and **b** rectal (n = 390) biopsies shows separate cluster for CD and non-IBD controls. **c** Volcano plot shows the DE analysis results from ileal samples and shows a total of 89 DE ncRNAs between CD and non-IBD controls **d** whereas in rectal biopsies, there were a total of 41 DE ncRNAs were identified **e**, **f** Hierarchical clustering in heatmap shows a clear separation of CD and non-IBD controls for 89 DE ncRNAs in ileal biopsies and 41 DE ncRNAs in rectal biopsies. **g** The log2FC of 130 DE ncRNAs obtained from both ileal and rectal biopsies are plotted. Each dot represents a CD specific DE ncRNA either in ileum or rectum. The ncRNA that are significant only in ileum, only in rectum, and both ileum and rectum are marked in dark red, light red and blue, respectively. **h** The CD specific DE ncRNAs with inverted effects in both ileum and rectum are plotted

Overall ncRNA transcriptomic profiles from ileal biopsies explained 34% and 12% of the variances with the first two PCs (Fig. 1a), whereas in rectal biopsies it only explained 16% and 7% variances (Fig. 1b), respectively. The first two PCs from the entire ncRNA transcriptomic profile showed that ncRNA levels have the potential by nature to discriminate CD from non-IBD controls (Fig. 1a, b). Further differential expression (DE) analysis identified a total of 89 DE ncRNAs in the ileum when comparing 274 CD cases to 71 controls. Of them, 62 were up-regulated in CD, while 27 were down-regulated (Fig. 1c). A similar comparison in rectal biopsies showed 41 DE ncRNAs when 329 CD cases were compared to 61 controls (Fig. 1d). Of those, 18 were up-regulated and 23 were down-regulated in CD. A hierarchical clustering of DE ncRNAs showcased two independent clusters representing CD and control groups. This pattern was observed for both ileum and rectal biopsies using the corresponding DE ncRNAs observed in each tissue separately (Fig. 1e, f). List of all FDR significant DE ncRNAs at FDR < 0.05 as well as the nominally significant DE ncRNAs *(P* < 0.05) for both ileal and rectal biopsies are provided in Additional file 4: Table S3. To further test whether the expression of CD specific ncRNAs are consistent across the different location of the intestine, we compared the log2FC of ncRNAs that are observed in both ileal and rectal biopsies. Of the 130 DE ncRNAs tested, 17 were shared in both the ileum and rectum, 87 were differentially expressed only in ileum, and 25 were differentially expressed only in the rectum (Fig. 1g, Table 2, Additional file 2: Fig. S2a). We determined whether the disease-specific expression of the 130 DE ncRNAs are expressed in the same direction or magnitude regardless of tissue type by comparing the log2FC of ileal DE ncRNAs to those in the rectum (Fig. 1g). Surprisingly, 88% (n = 114) of DE ncRNAs were expressed in the same direction regardless of tissue types, with a strong positive correlation of R = 0.69; *P* < 2.2e−16. The remainder (n = 16) were directionally inconsistent, with a strong negative correlation of R = -0.79; *P* < 2.2e−16 (Fig. 1h). These 16 ncRNAs showed unique, statistically significant differences when examined in both ileal and rectal samples amongst disease status. Similar to previous analysis [10], most of the DEncRNA found in our analysis were either antisense or lincRNAs.

**Table 2:** CD versus Controls DEncRNAs

Name	Ileum Log2FC	Rectum Log2FC	Ileum *P* value	Rectum *P* value	Significant
AC007228.9	1.296	1.457	1.24E−28	1.84E−26	Both
AC159540.1	1.001	1.095	3.80E−21	1.82E−24	Both
BCYRN1	− 1.068	− 0.691	7.34E−13	7.82E−06	Both
FAM225A	2.541	1.498	9.97E−41	1.41E−15	Both
MIR1244-2	1.374	1.058	1.95E−61	1.30E−30	Both
MIR1244-3	1.626	1.343	3.34E−58	1.81E−30	Both
MIR1244-4	1.586	1.199	1.15E−48	1.05E−27	Both
OVCH1-AS1	1.553	1.399	4.51E−71	9.01E−47	Both
RF00019	− 1.22	− 0.677	2.44E−17	6.35E−08	Both
RN7SL2	− 1.564	− 1.371	3.01E−47	1.81E−33	Both
RP11-143J24.1	1.863	1.625	1.59E−58	7.05E−38	Both
RP11-184E9.1	1.876	1.681	7.55E−51	5.40E−33	Both
RP11-566K8.2	− 1.096	− 0.521	9.39E−14	6.58E−07	Both
RP11-734K23.9	1.459	1.391	6.63E−34	1.67E−29	Both
RP11-79H23.3	1.16	1.503	4.32E−13	3.61E−14	Both
RP11-88H9.2	1.024	1.311	2.07E−12	1.95E−09	Both
RP11-90D4.2	1.63	1.036	5.87E−18	5.75E−11	Both
RN7SKP22	− 1.146	0.61	2.31E−11	3.17E−01	Both
AC007126.1	− 1.543	0.347	6.61E−17	4.94E−01	Ileal
AC016735.2	2.725	0.911	1.16E−15	6.08E−03	Ileal
AC017002.1	1.372	1.105	1.51E−22	6.09E−08	Ileal
AC023590.1	− 1.1	− 0.38	2.15E−13	1.12E−02	Ileal
AC068491.1	1.227	0.716	2.08E−25	1.39E−09	Ileal
AC114730.3	1.214	0.029	1.27E−05	7.37E−01	Ileal
AC133644.2	1.437	0.882	4.32E−29	5.63E−08	Ileal
AFAP1-AS1	1.036	0.55	7.10E−05	5.06E−04	Ileal
AP001046.5	1.392	0.863	5.35E−20	1.25E−07	Ileal
B3GALT5-AS1	1.123	− 0.39	4.89E−05	1.51E−02	Ileal
BLACAT1	1.007	0.549	9.38E−15	7.79E−04	Ileal
C12orf36	2.123	− 0.294	9.81E−14	2.86E−02	Ileal
C12orf61	1.062	0.495	9.32E−17	4.10E−06	Ileal
CDKN2B-AS1	− 1.023	− 0.071	1.28E−07	6.17E−01	Ileal
CTA-384D8.36	1.158	0.584	6.26E−19	1.15E−06	Ileal
CTB-127C13.1	− 1.135	1.492	2.87E−10	1.76E−03	Ileal
CTB-43E15.3	1.644	0.979	2.23E−13	3.68E−05	Ileal
CTB-61M7.2	4.619	2.916	2.63E−26	4.20E−10	Ileal
CTC-210G5.1	− 1.106	0.74	9.33E−09	5.42E−02	Ileal
CTC-490G23.2	3.255	− 0.056	5.64E−13	7.97E−01	Ileal
CTC-558O2.1	− 1.068	− 0.303	1.73E−11	3.84E−03	Ileal
CYTOR	1.512	0.979	1.57E−33	2.22E−17	Ileal
FAM225B	2.054	1.277	1.59E−28	1.75E−13	Ileal
HOTTIP	2.857	− 0.206	9.01E−08	1.80E−02	Ileal
HOXA11-AS1	2.247	− 0.056	1.06E−11	4.95E−01	Ileal
LINC00346	1.549	0.269	1.22E−20	3.39E−02	Ileal
LINC00479	− 1.255	− 0.295	2.71E−11	2.26E−01	Ileal
LINC01127	2.775	1.017	3.05E−25	7.24E−06	Ileal
LINC01272	2.847	0.756	2.02E−27	3.48E−05	Ileal
LINC01595	− 1.45	− 0.295	3.53E−06	4.40E−01	Ileal
MAP3K20-AS1	3.232	0.226	4.36E−24	2.03E−01	Ileal
NCRNA00114	1.273	0.73	6.18E−15	2.41E−05	Ileal
RN7SKP22	− 1.146	0.61	2.31E−11	3.17E−01	Ileal
RP11-109E12.1	1.913	1.492	2.76E−38	2.34E−28	Ileal
RP11-115D19.1	2.375	1.497	1.35E−18	3.75E−06	Ileal
RP11-116O18.1	− 2.224	1.449	4.06E−24	1.00E−03	Ileal
RP11-1334A24.6	1.019	0.835	2.80E−11	8.02E−08	Ileal
RP11-138C24.1	3.41	1.76	4.40E−17	1.41E−04	Ileal
RP11-1399P15.1	1.451	0.609	1.21E−17	1.92E−06	Ileal
RP11-178A10.1	1.832	0.461	6.06E−15	1.04E−02	Ileal
RP11-17H4.2	1.652	0.601	1.46E−19	7.40E−05	Ileal
RP11-203B7.2	− 1.435	− 0.392	7.11E−10	1.48E−01	Ileal
RP11-208N14.5	2.608	0.084	6.35E−17	5.53E−01	Ileal
RP11-213H15.3	3.262	1.333	3.58E−20	1.09E−04	Ileal
RP11-21C4.1	1.405	0.379	8.97E−15	3.49E−02	Ileal
RP11-304L19.3	1.042	0.476	2.03E−19	2.27E−07	Ileal
RP11-354P11.4	− 1.425	0.451	3.51E−15	3.90E−01	Ileal
RP11-380P13.1	− 1.079	− 0.411	4.27E−08	2.59E−02	Ileal
RP11-418I22.2	− 1.246	− 0.108	5.65E−11	6.75E−01	Ileal
RP11-419K12.2	1.874	0.697	2.15E−10	1.40E−02	Ileal
RP11-44K6.2	1.547	2.142	2.10E−12	2.91E−09	Ileal
RP11-478K15.6	− 1.52	− 0.382	7.82E−20	1.68E−02	Ileal
RP11-492E3.2	1.343	0.464	1.52E−17	5.09E−05	Ileal
RP11-515O17.2	− 1.001	− 0.028	4.70E−06	9.50E−01	Ileal
RP11-536O18.2	2.877	0.94	9.07E−24	1.90E−05	Ileal
RP11-638I2.9	1.214	0.598	9.50E−13	4.80E−04	Ileal
RP11-701P16.5	4.053	2.009	1.03E−20	2.54E−08	Ileal
RP11-806H10.4	1.168	0.851	3.83E−11	2.29E−06	Ileal
RP11-806L2.3	1.188	0.946	8.13E−19	2.35E−11	Ileal
RP11-817J15.3	− 1.166	0.673	1.32E−04	2.92E−02	Ileal
RP11-875H7.4	1.538	0.153	6.28E−18	2.30E−01	Ileal
RP11-94C24.13	− 1.125	− 0.369	4.25E−15	6.62E−03	Ileal
RP11-96M12.1	− 1.407	0.765	6.75E−10	7.57E−02	Ileal
RP13-497K6.1	− 1.08	− 0.408	1.97E−08	2.72E−02	Ileal
RP3-368B9.2	− 1.153	0.178	2.21E−07	6.30E−01	Ileal
RP4-724E13.2	− 1.297	− 0.66	1.26E−21	7.49E−08	Ileal
RP5-899E9.1	1.52	0.498	6.13E−23	2.26E−04	Ileal
SATB2-AS1	1.165	− 0.203	7.02E−07	2.12E−02	Ileal
SH3PXD2A-AS1	1.383	0.293	1.89E−11	2.30E−02	Ileal
SNHG9	1.229	0.959	8.91E−39	8.48E−18	Ileal
UCA1	1.151	0.296	1.51E−07	1.76E−01	Ileal
XXyac-YM21GA2.7	− 1.373	− 0.197	4.11E−12	3.64E−01	Ileal
RP11-116O18.1	− 2.224	1.449	4.06E−24	1.00E−03	Ileal
AC007126.1	− 1.543	0.347	6.61E−17	4.94E−01	Ileal
RP11-354P11.4	− 1.425	0.451	3.51E−15	3.90E−01	Ileal
RP11-96M12.1	− 1.407	0.765	6.75E−10	7.57E−02	Ileal
RP11-817J15.3	− 1.166	0.673	1.32E−04	2.92E−02	Ileal
RP3-368B9.2	− 1.153	0.178	2.21E−07	6.30E−01	Ileal
CTB-127C13.1	− 1.135	1.492	2.87E−10	1.76E−03	Ileal
CTC-210G5.1	− 1.106	0.74	9.33E−09	5.42E−02	Ileal
B3GALT5-AS1	1.123	− 0.39	4.89E−05	1.51E−02	Ileal
SATB2-AS1	1.165	− 0.203	7.02E−07	2.12E−02	Ileal
C12orf36	2.123	− 0.294	9.81E−14	2.86E−02	Ileal
HOXA11-AS1	2.247	− 0.056	1.06E−11	4.95E−01	Ileal
HOTTIP	2.857	− 0.206	9.01E−08	1.80E−02	Ileal
AC005392.1	3.255	− 0.056	5.64E−13	7.97E−01	Ileal
AC066593.1	0.146	− 0.545	5.63E−01	1.26E−03	Rectal
AC104135.4	0.894	1.445	7.86E−05	4.94E−07	Rectal
AP000345.1	− 0.779	− 0.765	5.37E−04	5.99E−04	Rectal
CTC-425O23.2	− 0.853	− 0.601	1.27E−13	4.39E−07	Rectal
CTD-2561J22.4	− 0.974	− 0.62	1.20E−11	2.92E−06	Rectal
DIO3OS	− 0.629	− 0.531	3.40E−08	8.76E−05	Rectal
HOXD-AS2	1.377	− 0.54	6.44E−09	1.51E−03	Rectal
LINC00923	− 0.401	− 0.553	7.70E−04	9.25E−06	Rectal
LL22NC03-2H8.5	− 0.897	− 0.692	9.65E−12	1.84E−11	Rectal
MIR145	− 0.501	− 0.612	3.12E−07	1.05E−06	Rectal
MIR3936	− 0.811	− 0.548	3.62E−16	1.42E−07	Rectal
MIR429	− 0.617	− 0.515	1.74E−07	1.06E−09	Rectal
RN7SKP127	− 1.108	− 0.656	1.07E−06	3.13E−04	Rectal
RN7SL3	− 0.663	− 0.875	4.39E−06	5.20E−08	Rectal
RP11-117B7.1	0.758	1.084	1.07E−19	1.41E−16	Rectal
RP11-125B21.2	0.268	− 0.733	2.21E−01	2.00E−05	Rectal
RP11-13K12.5	0.152	− 0.609	4.87E−01	1.95E−04	Rectal
RP11-283C24.1	1.872	1.69	1.26E−27	1.42E−19	Rectal
RP11-462G2.1	0.578	1.043	4.76E−04	5.23E−09	Rectal
RP11-527H14.2	0.834	1.34	3.74E−08	2.54E−09	Rectal
RP13-225O21.2	− 0.876	− 0.619	4.31E−14	2.75E−07	Rectal
RP13-516M14.10	− 0.678	− 0.531	2.53E−25	1.48E−14	Rectal
RP5-965G21.4	0.444	− 0.516	8.04E−03	2.26E−08	Rectal
U62631.5	− 0.901	− 0.541	3.15E−06	3.35E−03	Rectal
HOXD-AS2	1.377	− 0.54	6.44E−09	1.51E−03	Rectal

In total, there were 89 and 41 CD specific DE ncRNAs were identified in ileal alone and rectal alone biopsies, respectively. Significant column represents whether the CD specific ncRNA is significant in Ileal or rectal or in both

Next, to test our findings robustness and reliability, we compared our current results to our previous study [10], which contains a subset of ileal samples from the same RISK cohort. For this comparison, we excluded the matched ileal samples (n = 212) that were shared with our previous study, and in this subset analysis, 188 DE ncRNAs were observed. A direct log2FC comparison of ncRNAs expression in CD patients from both the studies showed a directionally consistent pattern with a strong positive correlation of R^2^ = 0.86; *P* < 2.2E−16, validating our methods and replicability (Additional file 2: Fig. S2b).

Taken together, these results show that changes in mucosal ncRNA levels are specific to CD and are most prevalent in the small intestine, but interestingly shows distinct changes in ncRNA signatures in the rectum. In contrast, from the 114-disease specific DE ncRNAs, we also found a small number of were miRNAs (ncRNAs with < 22 length of nucleotides), namely, MIR*-1244-1*, *MIR1244-2*, and *MIR1244-3*, regardless of tissue location.

### Pathways annotated to CD specific ncRNAs

The total ncRNA transcriptomic data appear to show larger CD-specific differences in the ileum than in the rectum. Based on this, it was not surprising that the TopGO pathway analysis on DE ncRNAs results showed more significant pathways hits in ileal (n = 136) (Additional file 2: Fig. S3a, b) than rectal biopsies (n = 36) (Additional file 5: Fig. S4a, b) between cases and controls (Additional file 5: Table S4 and Additional file 6: Table S5). However, there were 29 common pathways observed in ileal and rectal gene ontologies, including intracellular transport (GO:0,046,907) in the cellular component category that was annotated by *RN7SL2*.

### Behavior-specific DE ncRNAs in intestinal biopsies

**Fig. 2:**
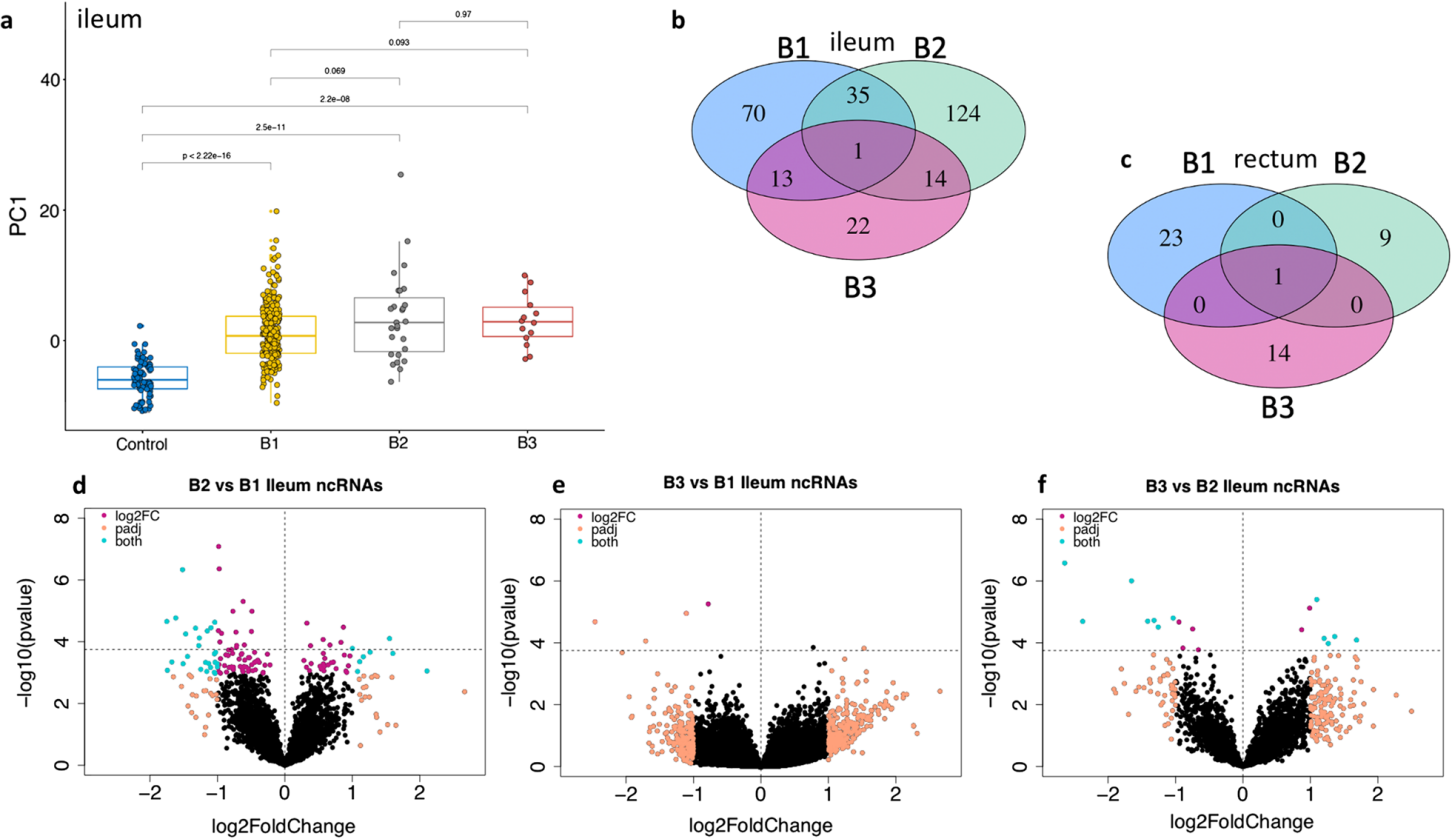
Crohn’s disease behavior DE ncRNAs. **a** Using n = 89 DE ncRNAs from CD versus Controls in ileal samples, Principal Components were calculated and the PC1 was used to visualize clustering of Montreal classifiers. **b** The DE analysis in ileal samples based one Montreal classifications reveals disease behavior specific ncRNAs and the number of DE ncRNAs in each comparison is plotted in Venn diagram **c** similar comparison in rectal biopsies was performed and the number of DE ncRNAs in each comparison is provided. **d**−**f** Inter-CD Montreal classifiers comparison results are provided in volcano plot, showing significant DE ncRNAs were observed in a pairwise comparison in only ileal biopsies

Next, we examined if PC1 of 130 DE ncRNAs that were observed between CD and controls were able to differentiate Crohn’s disease behaviors (B1, B2 and B3). As expected, the PC1 had a potential to differentiate one from the others (Fig. 2a). Therefore, we further extended our analysis based on CD disease behaviors. First, we compared the expression of ncRNAs among individual CD behavior group against controls in both ileal and rectal biopsies and revealed ncRNAs specific to distinct CD behavior groups. We noticed more DE ncRNAs in ileum; B1 (n = 70), B2 (n = 124) and B3 (n = 22) (Fig. 2b, Additional file 7: Table S6) than in the rectal biopsies; B1 (n = 23), B2 (n = 9) and B3 (n = 14) (Fig. 2c, Additional file 8: Table S7).

Similarly, the comparison among CD disease behavior groups from inflammatory (B1) to stricturing (B2) to penetrating (B3) showed an increased pattern of the variance explained 32%, 35%, 45%, respectively (Additional file 2: Fig. S5 a−g). The DE analysis among B1, B2 and B3 showed a similar tread, which is an ileal-centric nature with more DE ncRNAs were observed in ileal B2 versus B1 (n = 35), B3 versus. B1 (n = 13) and B3 versus. B2 (n = 14) than was found in the rectum (Additional file 9: Table S8; Fig. 2c). Interestingly, all DE ncRNAs observed in B3 versus. B1 (Fig. 2e) were also observed in B2 versus B1 (Fig. 2d) and B3 versus B2 (Fig. 2f) comparisons, potentially demonstrating certain CD characteristics that may be present across B1, B2, and B3. Notably, most of them were antisense or lincRNAs types of non-coding elements (Additional file 2: Fig. S6a, b). Similar comparison in rectal biopsies showed no DE ncRNAs to be statistically significant. Thus, our results indicate that a set of ncRNAs in ileal biopsies reflects the Montreal CD disease behaviors, whereas such a pattern was not observed in rectal biopsies of CD patients.

### Inflammation and disease location-specific ncRNAs in CD

**Fig. 3:**
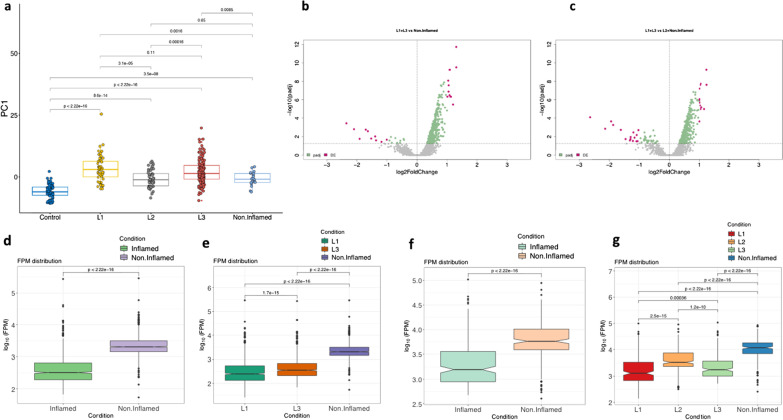
Crohn’s disease location DE ncRNAs. **a** Using n = 89 DE ncRNAs from CD versus controls in ileal samples, principal components were calculated and PC1 was used to visualize clustering of inflammation status on different disease location. **b** DE analysis compared using two groups. One was comprised of: L1 (51) + L3 (147) representing Inflamed, n = 198, versus non-inflamed (20), which resulted in n = 31 DE ncRNAs. The second comparative group consisted of L1 (n = 51) + L3 (n = 147) representing Inflamed, (n = 198), versus non-inflamed (47) (20 + L2 (27), resulting in 21 DE ncRNAs. **c** The volcano plots representation of DE analysis for both the comparisons. **d**, **e** To represent the DE ncRNAs according to location and inflammation status, normalized by log10(FPM) of 31 DE ncRNAs obtained from L1 + L3 ileal inflamed groups versus non-inflamed ileal groups (without L2) in CD patients, are compared in boxplots; similar comparison was made with 21 DE ncRNAs obtained from L1 + L3 ileal inflamed groups versus non-inflamed + L2 ileal groups in CD patients (**f**, **g**)

Inflammation is often a visible hallmark signature of CD, thus we next examined whether the expression of ncRNAs in the inflamed group of CD patients could distinguish them from the non-inflamed groups and furthermore, the location of disease. We used two groups that are assigned by physicians based on i) inflammatory status (inflamed vs. non-inflamed), and ii) disease (inflammation) location such as ileal-centric (L1), colonic (L2), and ileocolonic (L3). We tested whether the PC1 obtained from 130 CD specific DE (Fig. 1) can differentiate inflammatory status and disease location in CD patients. Overall, PC1 obtained from ileal biopsies (Additional file 2: Fig. S7a) showed significant differences between inflamed and non-inflamed/controls samples than rectal biopsies (Additional file 2: Fig. S7b). Especially PC1, as it largely differentiated the CD patients with L1 and L3 ileal inflamed disease locations from controls (*P* < 2.2E−16), as compared to L2 CD patients with colon inflamed disease location (*P* < 2.2E−14) or patients with non-inflamed sites (*P* < 3.5E−08) groups (Fig. 3a).

Therefore, we further subjected the CD patients to identify DE ncRNAs specific for inflammation status and location of the disease, which are classified through physician’s clinical assessment. Since this study is primarily focused on CD, where the disease largely occurs in the ileum, we restricted this analysis to only ileal biopsies. In order to identify the ncRNAs specific for disease locations, the L1 and L3 CD patients (n = 198) were combined as the inflamed group and then we compared with non-inflamed ileal CD patients (n = 20) alone and then to non-inflamed + L2 (n = 76) groups together (Additional file 2: Fig. S8a−c), keeping in mind that the L2 CD patients were inflamed only in the colonic location, not in the ileum. Our DE analysis on the ileal biopsies showed 21 DE ncRNAs for L1 + L3 versus non-inflamed (Fig. 3b), and 31 DE ncRNAs for L1 + L3 versus non-inflamed + L2 (Fig. 3c) (Additional file 10: Table S9). A total of 10 DE ncRNAs were shared in both comparisons (Fig. 3d). Likewise, using log normalized FPM (fragments per million) of 21 DE ncRNAs showed better differentiation between the inflamed (L1 + L3) non-inflamed groups (Fig. 3e) rather than the other comparison with 31 DE ncRNAs (Fig. 3f), which incorporated L2 samples into non-inflamed group. Further, the FPM analysis on specific inflammation location showed both L1 and L2 groups as being similar, while L2 and non-Inflamed groups as more closely related (Fig. 3g-h). Using these results in comparing disease inflammation and location status, ncRNA transcriptomic profiles of L2 CD patients were more like CD patients with non-inflamed ileal disease location than inflamed ones (L1, L3).

### DE ncRNAs *RN7SL2, mir-1244-2,3,4*

**Fig. 4:**
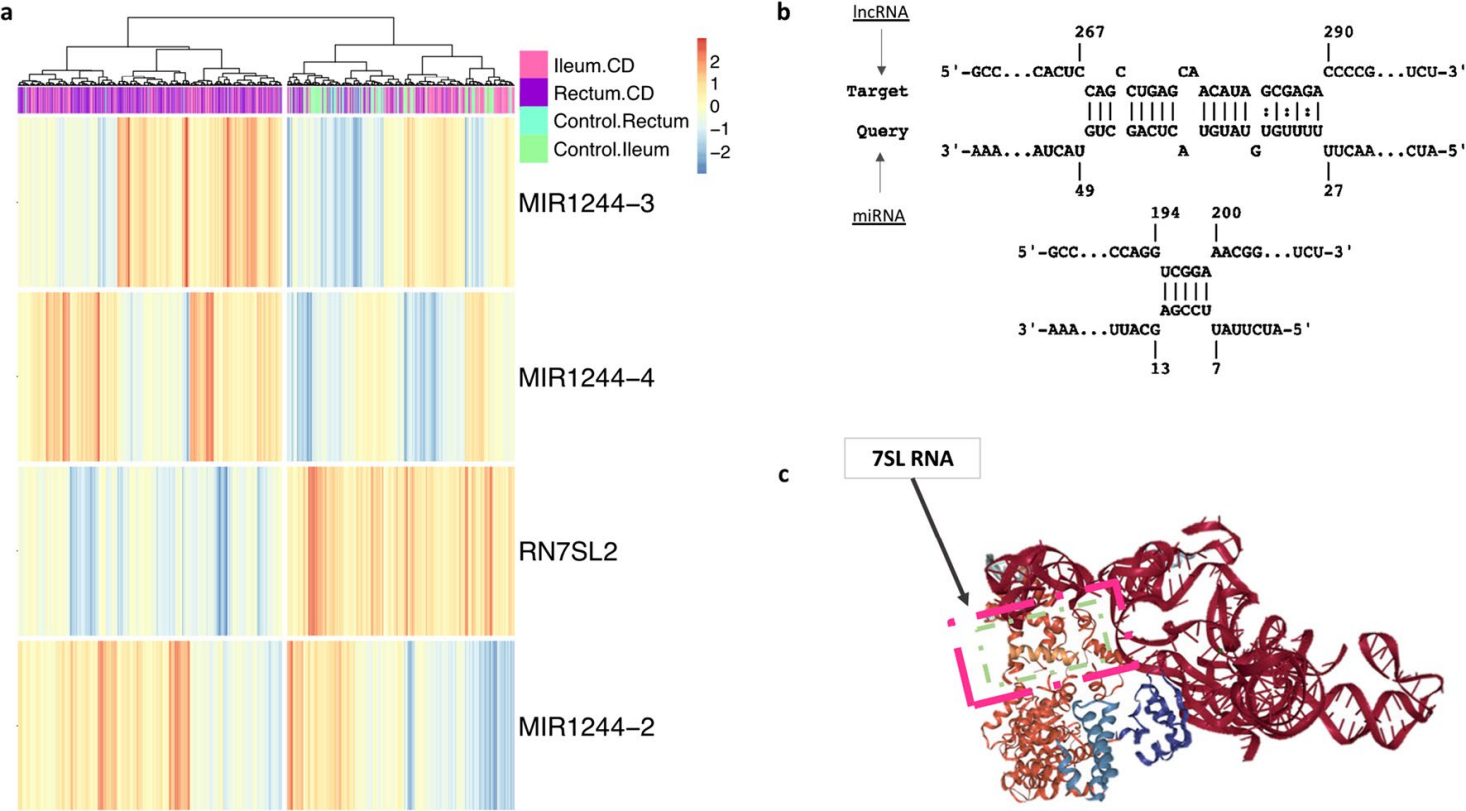
Hypothesized ncRNA-miRNA Interactions. **a** The ncRNAs’ RN7SL2, mir1244-2, mir1244-3, and mir1244-4 log2FC in CD versus controls amongst ileal and rectal samples were calculated and combined to generate one heatmap differentiating CD versus Controls by Ward-D clustering. **b** Using IntaRNA, Freiburg RNA tools, thermodynamics and kinematics of the predicted binding affinities are shown below amongst one target, RN7SL2, a lncRNA, & three miRNAs, mir1244-2, mir1244-3, and mir1244-4: A: [RN7SL2]: ‘268’ -- ‘289’ & [miRNA-1244-x]: [‘28’ -- ‘48’]. The second predicted interaction located at B: [RN7SL2]: ‘195’ -- ‘199’ & [miRNA-1244-x]: [‘8’ -- ‘12’]. **c** The 3-D conformational location of RN7SL2, often the 7SL 1244-x]: [‘8’ -- ‘12’]. The 3-D conformational location of RN7SL2, often the 7SL component of the SRP (Signal Recognition Particle) is shown

miRNAs have been observed to regulate multiple facets of gene expression including other non-coding RNAs, and are known to be dysregulated during CD [24], yet the mechanisms remain unclear. Of interest is the down regulation of *RN7SL2* by *mir-125b* to control cell death [25]. In our analysis, we found an increase in the levels of *mir-1244-2,3,4* and a decrease in the levels of *RN7SL2* in CD versus controls (FDR significance, but not in log2FC) (Fig. 4a). Using IntaRNA to test for molecular interactions amongst *RN7SL2* and *miRNA-1244-x* (*2,3,4*), we obtained six possible predicted conformations with stable base pairing (Table 3). For two of these possible interactions, one had complementary base pairing for the miRNA-ncRNA interaction at *RN7SL2*, nucleotides 268–289 with *miRNA-1244-2,3,4* at nucleotides 28–48, while the second predicted interaction was *RN7SL2*, nt 195–199 with *miRNA-1244-2,3,4* at nts 8–12. The Δ°G values of these interactions suggest stable binding and schematically represented in Fig. 4b, along with a more realistic molecular model generated by using SRP RNA structures based on ribosomal RNA interactions (Fig. 4c) [26]. Taken together, these results suggest that changes in miRNA levels during CD have physiological impacts that can change cellular function and potentially alter disease outcomes, with RN7SL2 being a potential candidate for targeted therapy.

**Table 3:** Predicted binding affinities of RN7SL2 lncRNA with mir-1244-2, mir-1244-3, mir-1244-4 miRNAs

lncRNA	P-lncNRA	miRNA	P-miRNA	Energy (kcal/mol)	H-Energy	UE−lncRNA	UE-miRNA
*RN7SL2*	268–289	*miRNA-1244–4*	28–48	− 5.52	− 16.3	5.16	5.65
*RN7SL2*	195–199	*miRNA-1244–4*	8–12	− 3.64	− 5.4	1.6	0.19
*RN7SL2*	195–199	*miRNA-1244–2*	8–12	− 3.64	− 5.4	1.6	0.18
*RN7SL2*	195–199	*miRNA-1244–3*	8–12	− 3.64	− 5.4	1.6	0.18
*RN7SL2*	268–289	*miRNA-1244–2*	28–48	− 3.32	− 14.1	5.16	5.64
*RN7SL2*	268–289	*miRNA-1244–3*	28–48	− 3.32	− 14.1	5.16	5.64

*P-lncRNA* position of ncRNA, *P-miRNA* position of miRNA, *H-Energy* hybridyzation energy, *UE-lncNRA* unfold entergy lncRNA, *UE-miRNA* unfold entergy miRNA

### ncRNA as a potential tool to predict disease status, disease behaviors and disease location in IBD

**Fig. 5:**
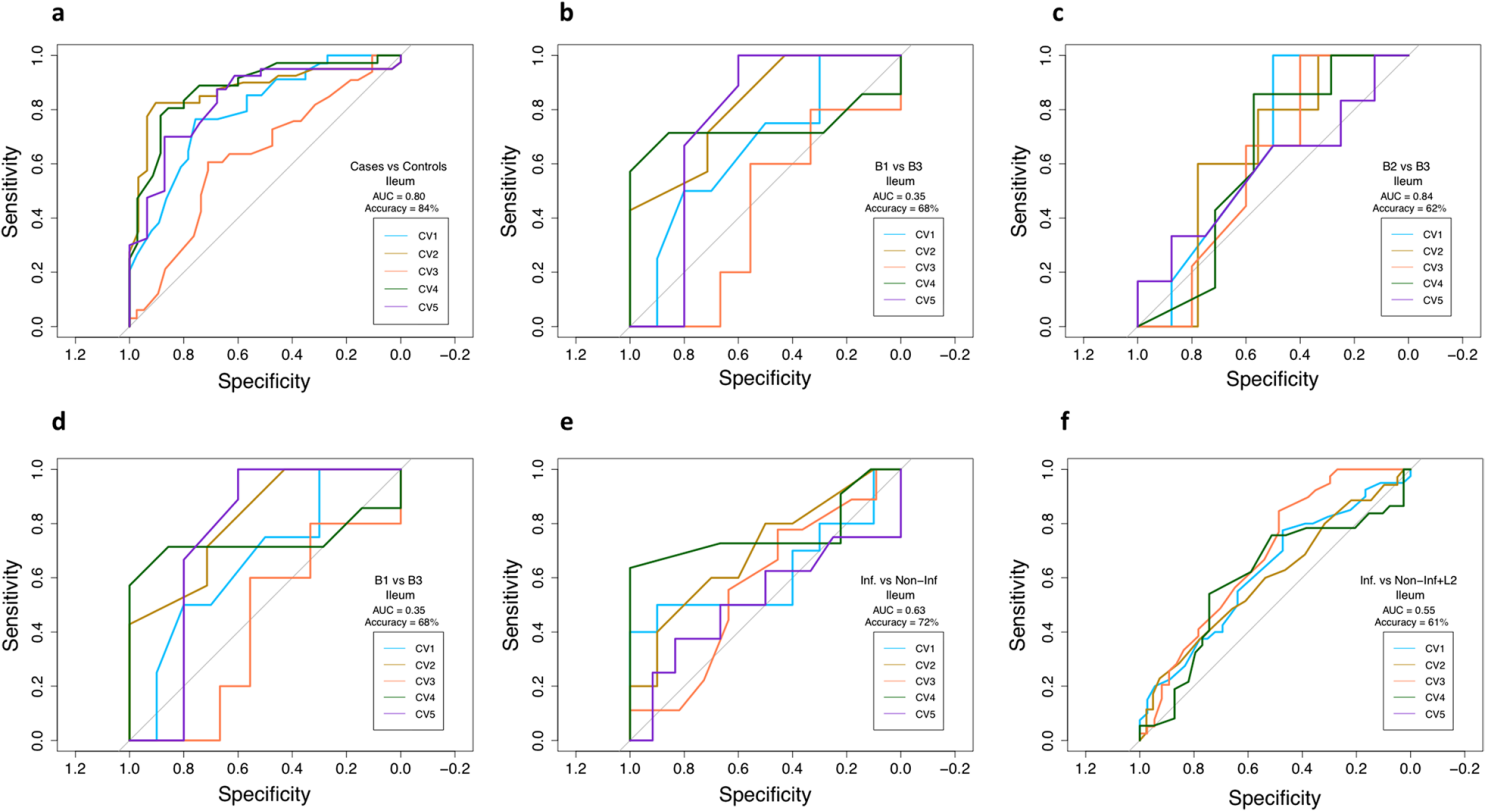
Predicting ability of ncRNA for disease status, disease behaviors and inflammation location. Using n = 13,777 ncRNAs with at least base mean > 10, we tested to predict the disease status, disease behavior and disease location or inflammation in CD. The average Accuracy and AUC of all 5 cross-validations is denoted on the figure(s). **a** Predicting ability of ncRNA for disease status, AUC and accuracy was calculated for CD **b** Predicting ability of ncRNA for disease behavior (B2 from B1), AUC and accuracy was calculated for B1, **c** Predicting ability of ncRNA for disease behavior (B3 from B1), AUC and accuracy was calculated for B2, **d** Predicting ability of ncRNA for disease behavior (B3 from B2), AUC and accuracy was calculated for B3, **e** Predicting ability of ncRNA for disease inflammation (inflamed vs. non-inflamed), AUC and accuracy was calculated for non-inflamed and **f** Predicting ability of ncRNA for disease inflammation (inflamed vs. non-inflamed + L2), AUC and accuracy was calculated for non-inflamed. A five-fold cross validation (CV) was performed across all the comparisons

Lastly, we tested the accuracy of these non-coding elements to predict disease from controls, disease behavior, and disease inflammation in ileal biopsies through RandomForest [27] approach. To test whether ncRNAs serve as a potential index to predict disease status, we used the entire dataset of both CD and CTRLs. The specificity and sensitivity of the modeling showed an average AUC of 0.80 with 84% accuracy, reflecting the robustness of these DEncRNAs to decipher CD versus controls (Fig. 5a). Whereas, in terms of disease behavior, due to our dataset being composed of limited sample size in B2 and B3 when compared to B1, we arbitrarily down sampled the larger dataset in each comparison with respect to the smaller comparative dataset. Therefore, to predict the B2, and B3, from B1, we randomly down sampled B1 to mitigate sample bias. With this, our model predicted B2 from B3 with a mean AUC of 0.84 and 80% accuracy (Fig. 5c), B2 from B1 with 0.72 AUC and 62% accuracy (Fig. 5b), and B3 from B1 with 0.68 AUC and 68% accuracy (Fig. 5d). Likewise, in comparing inflammatory status, the inflamed samples were down sampled with respect to non-inflamed samples. Our model showed better prediction to non-inflamed (without L2) from inflamed with 0.63 AUC and 72% accuracy (Fig. 5e). Interestingly, a poorer prediction was observed when non-inflamed and L2 were tested against inflamed displaying 0.55 AUC and 61% accuracy (Fig. 5f). Details of sample sizes in each comparison and fivefold cross-validation prediction results for disease status, disease behavior and inflammation status are provided in Additional file 11: Table S10, Additional file 12: Table S11, Additional file 13: Table S12, Additional file 14: Table S13, Additional file 15: Table S14, Additional file 16: Table S15.

## Discussion

While changes in ncRNA during CD have been documented [6, 10, 11, 28, 29], little is known about the role of ncRNAs in different location of the intestine. At diagnosis, the location of the diseased mucosal biopsy obtained through colonoscopy often determines diagnosis of IBD subtypes. Inflammation in the ileum is most common for CD whereas rectal inflammation is common for UC. As often is the case however, CD can manifest at multiple locations along the intestine. Using the same IBD patients from the RISK cohort, our previous transcriptomic analysis on the protein-coding genes and a combined analysis with genotypes (eQTL) of both UC and CD in different tissues types, ileum and rectum [30] showed that the transcriptomic and eQTL signatures are distinct to disease characteristics. Here in, we have taken a similar approach and have applied it to profiling ncRNAs in conjunction with location and behavior specific. In doing so we have expanded on previous analysis by including a larger number of CD patients. Importantly, we have shown that ncRNA changes correlated with clinical subtypes originally diagnosed by the physician, and thus it can potentially be applied as a tool to categorize CD disease and corresponding inflamed disease location through ncRNA profiling.

We previously observed that the gene signatures associated with development of complications in CD are ileal specific and often associated with genes involved in producing extracellular matrix (ECM) when progressing from B1 to B2 forms of CD [12]. In our current study, we found the highest degree of DE ncRNA in the ileum, consistent with ileal-centric disease, but also observed multiple DE ncRNAs within the ileum and rectum associated with the ECM. For example, the ncRNA, *AC016735.2*, has been observed to regulate *COLIA1* and *COLIA2* whose function involves mediation of collagen organization, a prevalent component of the stromal ECM [31], and we found that *AC016735.2* was the most differentially expressed in B1 and B3 disease. Since the intestinal epithelium requires properly functioning ECM in order to establish a functional barrier from luminal contents to prevent adverse immune responses [32], the ability to track early signs of collagen dysregulation by monitoring ncRNA levels may be an important diagnostic tool and potential target for therapy. The involvement of *AC016735.*2 in other intestinal diseases such as gastric cancers [31], suggests imbalances in this molecules function may play a negative role in IBD outcomes. Thus, by mapping such changes through ncRNA profiling and the location where they are taking place, we have shown a ncRNA profile for CD patients and provided further insights into potential epigenetic sources of disease manifestation i.e. ncRNA dysregulation, within the mucosa.

In a Danish cohort of 213 CD patients, it was observed that individuals with L1: ileal site of disease manifestation and B2: stricturing behavior exhibited the highest risk for surgical intervention [33]. Our results, using CD classifications, show individuals with B2 CD had the lowest correlation coefficient, independent of tissue type, in comparison to B1 and B3. Likewise, L1: ileal, forms of CD also exhibited the lowest correlation coefficients in both the rectal and ileal datasets. Patients with B2-CD and L1: ileal site of disease localization was the most distinct in terms of transcriptomic profiling utilizing ncRNAs. Therefore, we demonstrate that the levels of non-coding genetic elements reflect distinct changes in CD patients that correlate with other clinical indicators and indexes of diagnosis, giving further validity to their potential use as biomarkers.

Consistent with our previous reports [10] and Braga-Neto et al. [34], we detected *LINC01272* to be 1.65 × DE in increased quantities, ~ 2.9 log2FC, in CD versus controls, in both tissue types. Across the ileum and rectum samples, multiple DE ncRNAs such as, *OVCH1-AS1*, *RP11-143J24.4*, and *RP11-184E9.1*, showed increased expression in CD versus controls. Notably, *RN7SL2* was the only down-regulated DE ncRNA that was observed in both the rectum and ileum displaying significantly lower levels in diseased CD samples. *RN7SL2* serves as a 7SL RNA molecule and serves to scaffold the formation of a cytoplasmic ribonucleoprotein complex, Signal Recognition Particle (SRP). This type of non-coding ribonucleoprotein (ncRNP) is conserved across multiple species and the mammalian versions of SRP are comprised of six proteins and one RNA molecule, RN7SL2 [35]*.*

Notably, *RN7SL2* or *RNSRP2* was downregulated in both the ileum and rectum biopsies of pediatric CD patients in comparison to controls. It was the only ncRNA across the assessment panel to be statistically significant and observed across disease status, CD versus controls, and disease behavior (B1, B2), in both the ileal and rectal mucosa. This suggests that certain non-coding genetic elements associated with CD may be detected across different tissues and sites of disease manifestation with similar DE trends. *RN7SL2* functions involve mediation of secretory proteins into the lumen of the endoplasmic reticulum (ER), sometimes via co-translational-insertion [36]. Also, there is an additional conformation of 7SL-RNA (*RN7SL2)* that forms a propeller-secondary structure with two hairpins being converged by a tetranucleotide bulge loop at its 5’-end that increases its topological efficiency allowing up-regulated activation of pol-III transcription [37]. Thus, changes in this molecule could potentially impact protein processing through the ER, export to the cell surface, vesicle release as microparticles, and even affect the translation of proteins at the ribosome by manipulating the transcription activities of pol-III and its transcribing of rRNA and tRNA. However, how these changes in RN7SL2 levels during CD drives the onset and/or progression of CD still needs further analysis.

Collectively, our Random Forest prediction results show that ncRNAs could be used as a tool to discriminate CD from non-IBD with great accuracy. Nevertheless, our sample sizes are limited for disease behavior and inflammation statuses to achieve a better prediction model. With these results, we believe that clinical disease classifiers further support the utility of non-coding elements as potential biomarkers, prognostic tools, and pharmaceutical targets for therapy.

Taken together, our studies have revealed a clear change in the levels of ncRNAs in the mucosa during distinct phases of CD that correlate well with clinical classifiers. The predominant changes were ileal-specific signatures that likely involved changes in both the ECM and factors that regulate protein function at the ribosome, ER and nucleus. ncRNA changes are thus promising indexes of disease behavior and could potentially serve as therapeutic targets for treatment of distinct stages of CD.

## Conclusion

Our study has shown that ncRNA in the intestinal mucosa change during CD in the ileum and the rectum and correlate well with clinical indicators, but that the largest percentage of those changes occurred in the ileal tissues, reflecting the ileal-specific nature of CD. Since these signatures appear to correlate well with severe disease and location, they are most likely strong indicators of disease status. Although it is unclear from our analysis if the changes in ncRNA levels are in fact cellular repair measures or further contributing to mucosal injury, the dysregulated levels of ncRNA in mucosal tissue of CD patients suggests they play a role in CD and might have clinical utility in aiding in early identification and characterization of disease progression.

## Supplementary Information

**Additional file 1: Table S1.** List of fifteen types of non-coding RNAs categorization based upon their length of nucleotides.

**Additional file 2: Fig. S1.** Overall Workflow of Analysis: Differential expression analysis was performed on n = 345 ileal and n = 390 rectal Bulk-RNA-later biopsies using Illumina deep-RNA sequencing in a single batch. The reference panel utilized was Hg38 with STAR package used for alignment of 58,381 transcripts, of which n = 20,779 were ncRNAs. EdgeR was used for differential expression of these ncRNAs in three comparisons: Disease Status, Disease Behavior, and Disease Inflammation. For each analysis, the total number of DE ncRNAs for ileal and rectal datasets is represented. **Fig. S2.** **a** The log2FC obtained from case-control analysis of all the 20,779 ncRNAs were compared between ileal and rectal biopsies. Each point is a ncRNA expression and they colored based on tissue- or disease-specific. **b** Using a subset of newly diagnosed CD patients (n = 133) from the ileal dataset (n = 345), the DE analysis results were compared with our previous study (Haberman et al. 2018) results performed on the same RISK cohort. The strong positive log2FC results shows the reliability and replicability of our analysis. **Fig. S3.** Gene Ontology Analysis of Crohn’s Disease versus Controls DE ncRNAs in Ileal Biopsies: Using TopGO, gene ontology analysis was conducted on n = 89 DE ncRNAs in ileal biopsies. The results displayed significant, FDR < 0.05, hits in (**a**) cellular components (n = 79), and **b** biological processes (n = 21). **Fig. S4.** Gene Ontology Analysis of Crohn’s Disease versus Controls DE ncRNAs in Rectal Biopsies: Using TopGO, gene ontology analysis was conducted on n = 89 DE ncRNAs in rectal biopsies. The results displayed significant, FDR < 0.05, hits in **a** cellular components (n = 12), and **b** biological processes (n = 17). **Fig. S5.** Principal Components and Volcano Plots of DE ncRNAs based on Crohn’s Disease Behavior: The PCs were calculated using entire list of ncRNA (n = 20,779) and first two PCs were plotted. Each point represents a subject and they are clearly separating the CD disease severity groups from controls across **a** B1 versus controls, **b** B2 versus Controls, and **c** B3 versus Controls, in ileal biopsies. The volcano plots of DE analysis show DE ncRNAs were identified across d B1 versus controls (n = 70), e B2 versus controls (n = 124), f and B3 versus controls (n = 22) comparisons. **Fig. S6.** Types of ncRNAs observed in Crohn’s Disease Behavior DE ncRNAs: Sankey plots of DE ncRNAs observed in ileal samples by comparing **a** B2 versus B1 (n = 35), and **b** B3 versus B2 (n=14) were correlated with protein coding genes (n=18,927) from the same dataset. Those pairs of ncRNA-mRNA with correlations > 0.50 were deemed significant. Using these correlation pairs, the relationship of ncRNAs and mRNAs were sequestered based on CIS, same strand, or TRANS, different strands. The coding strand is represented by (−) and non-coding strand by (+). **Fig. S7.** Principal Component Analysis of CD patients available with Inflammation Status ncRNAs: The disease inflammation location classifiers: L1, L2, L3, were used to classify the inflammation status. Any CD patients had disease at L1, L2 and L3 were considered as inflamed and the rest were considered as non-inflamed samples. The first two PCs were plotted to see any clusters based on inflammation status in (**a**) ileal biopsies (n=345) and (b) rectal (n = 390) rectal biopsies. **Fig. S8.** Principal Component Analysis of Inflammation Location ncRNAs: Using all n = 20,779 ncRNAs, princomp was used to extrapolate PCs in order to visualize trends amongst disease inflammation location groups in entire CD patients. PCs were calculated for inflamed versus non-inflamed samples a Both L1 + L3 (n = 198) CD patients with ileal disease were considered as inflamed group and compared against the non-inflamed group (n = 20 **b**), and non-inflammed + L2 (n = 76) group, because the L2 CD patients are inflamed in colonic region (**c**).

**Additional file 3: Table S2.** The cohort Metadata Chi-squares: Chi-squared of patient characteristics such as sex, age, disease type, disease behavior, and inflammatory status in both ileal (n = 345), and rectal (n = 390) datasets.

**Additional file 4: Table S3.** The case-control DE analysis results on ileal (n = 345) and rectal (n = 390) biopsies, by comparing disease status (CD = 274 vs. CTRL = 71) in ileal and rectal (CD = 329 vs. CTRL=61) datasets. All FDR significant DEncRNAs and nominally significant (P < 0.05) obtained from both ileal (n=3520) and rectal (n = 1447) are provided Ileum Gene Ontology: Using TopGO with input of 89 DEncRNAs in ileal samples from CD versus controls, 136 pathways were observed. Of which, 36 were molecular function, 79 cellular components, and 21 biological processes.

**Additional file 5: Table S4.** Ileum Gene Ontology: Using TopGO with input of 89 DEncRNAs in ileal samples from CD versus controls, 136 pathways were observed. Of which, 36 were molecular function, 79 cellular components, and 21 biological processes.

**Additional file 6: Table S5.** Using TopGO with input of 41 DEncRNAs in rectal samples from CD versus controls, 36 pathways were observed. Of which, 7 were molecular function, 12 were cellular, and 17 biological processes.

**Additional file 7: Table S6.** Ileum Disease Behavior DEncRNAs. DE analysis results for ileal (n = 345) biopsies by comparing disease behavior against controls and among CD disease behaviors. All FDR significant DEncRNAs with log2FC > 1 and nominally significant (P < 0.05) are listed, B1 versus controls (n = 70), B2 versus controls (n = 124), and B3 versus controls (n = 22).

**Additional file 8: Table S7.** Rectum Disease Behavior DEncRNAs: DE analysis results for rectal (n = 390) biopsies by comparing disease behavior against controls and among CD disease behaviors. All FDR significant DEncRNAs and nominally significant (P < 0.05) are listed, B1 versus controls (n = 23), B2 versus controls (n = 9), and B3 versus controls (n = 14).

**Additional file 9: Table S8.** Intra-Disease Behavior DEncRNAs: DE analysis results for ileal (n = 345) biopsies by comparing among disease behaviors. All FDR significant DEncRNAs and nominally significant (P < 0.05) are listed, B2 versus B1 (n = 35), B3 versus B1 (n = 13), and B3 versus B2 (n = 14).

**Additional file 10: Table S9.** Inflammation and disease location specific DEncRNAs. DE analysis results for ileal (n = 345) biopsies by comparing disease inflammation status, non-inflamed, inflamed, against controls and within-CD. All FDR significant DEncRNAs and nominally significant (P < 0.05) are listed. Differential expression was compared using groups comprised of: L1 + L3 ileal Inflamed group, (n = 198) versus non-inflamed ileal group (20), identified 31 DEncRNAs, and the same inflamed group versus non-inflamed (47) (20) + L2 (27), identified 21 DEncRNAs.

**Additional file 11: Table S10.** Using RandomForest, n = 345 ileal patients were randomly split into equal number of samples, n = 71 controls and n = 71 CD. These were further aliquoted into test and train datasets for cross-validation of AUC with n = 13,777 ncRNAs, base mean greater than n > 10.00. The input included n = 71 CD and n = 71 controls used to test disease versus controls status by evaluating model and test for accuracy based on sensitivities and specificity.

**Additional file 12: Table S11.** Using RandomForest, n = 274 ileal patients were randomly split into equal number of samples, n = 14 B1 and n = 14 B2. These were further aliquoted into test and train datasets for cross-validation of AUC with n = 13,777 ncRNAs, base mean greater than n > 10.00. The input included n = 14 B1 and n = 14 B2 used to test disease versus controls status by evaluating model and test for accuracy based on sensitivities and specificity.

**Additional file 13: Table S12.** Using RandomForest, n = 274 ileal patients were randomly split into equal number of samples, n = 14 B2 and n = 14 B3. These were further aliquoted into test and train datasets for cross-validation of AUC with n = 13,777 ncRNAs, base mean greater than n > 10.00. The input included n = 14 B2 and n = 14 B3 used to test disease versus controls status by evaluating model and test for accuracy based on sensitivities and specificity..

**Additional file 14: Table S13.** Using RandomForest, n = 274 ileal patients were randomly split into equal number of samples, n = 14 B1 and n = 14 B3. These were further aliquoted into test and train datasets for cross-validation of AUC with n = 13,777 ncRNAs, base mean greater than n > 10.00. The input included n = 14 B1 and n = 14 B3 used to test disease versus controls status by evaluating model and test for accuracy based on sensitivities and specificity.

**Additional file 15: Table S14.** Using RandomForest, n = 274 ileal patients were randomly split into equal test and train datasets for cross-validation of AUC with n = 13,777 ncRNAs, base mean greater than n > 10.00. The input included n = 20 non-inflamed and n = 20 inflamed (L1 = 10 and L3 = 10) used to test disease inflammation status by evaluating model and test for accuracy based on sensitivities and specificity.

**Additional file 16: Table S15.** Using RandomForest, n = 274 ileal patients were randomly split into equal test and train datasets for cross-validation of AUC with n = 13,777 ncRNAs, base mean greater than n > 10.00. The input included n = 76 non-inflamed (+ L2 = 56) and n = 76 inflamed (L1 = 38, L3 = 38) used to test disease inflammation status by evaluating model and test for accuracy based on sensitivities and specificity.

## Data Availability

The ileal bulk-biopsy RNA sequencing data included in this study have been deposited in the Accession PRJNA594730 (https://www.ncbi.nlm.nih.gov/bioproject/PRJNA594730). The rectal bulk-biopsy RNA sequencing data is deposited in the GEO series accession number GSE117993. Analyses accompanied with ncRNA annotations was conducted using GENCODE v28 (HG38) (https://www.ncbi.nlm.nih.gov/assembly/GCF_000001405.26/) and Ensembl (http://useast.ensembl.org).

## Abbreviations

IBD: Inflammatory bowel disease
CD: Crohn’s disease
DE ncRNAs: Advanced Diabetic Eye Disease
FDR: False discovery rate
log2FC: Log-2fold change
p. adj: Adjusted *p* value after multiple test corrections
ROC: Receiver operator characteristic curve
AUC: Area under curve
