# Supplementary figures and images for: Profiling non-coding RNA levels with clinical classifiers in pediatric Crohn’s disease
Source: BMC Med Genomics. 2021 Jul 29;14:194. doi: 10.1186/s12920-021-01041-7 (PMC8323253; doi:10.1186/s12920-021-01041-7)

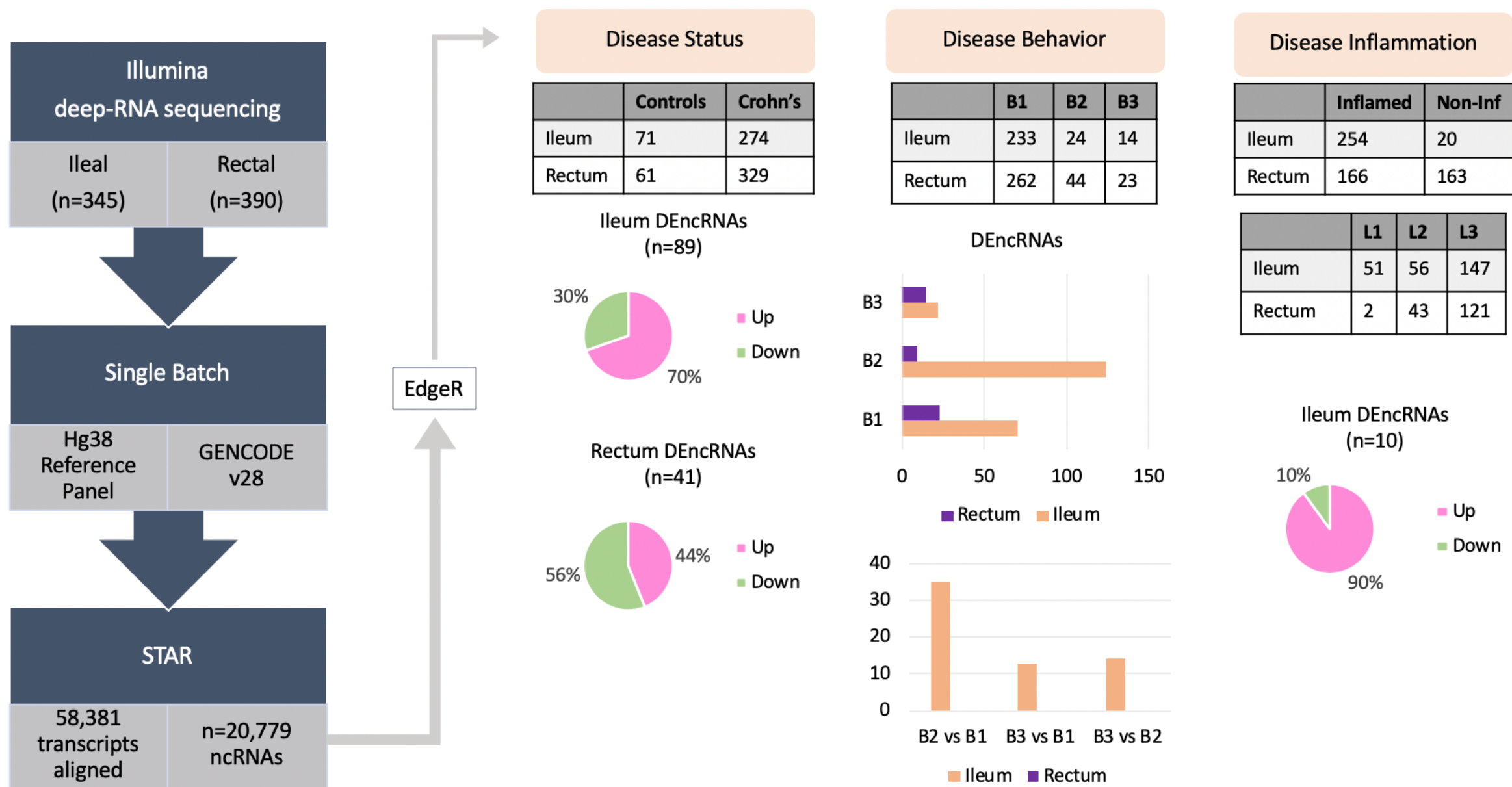

**Figure S1**

2a

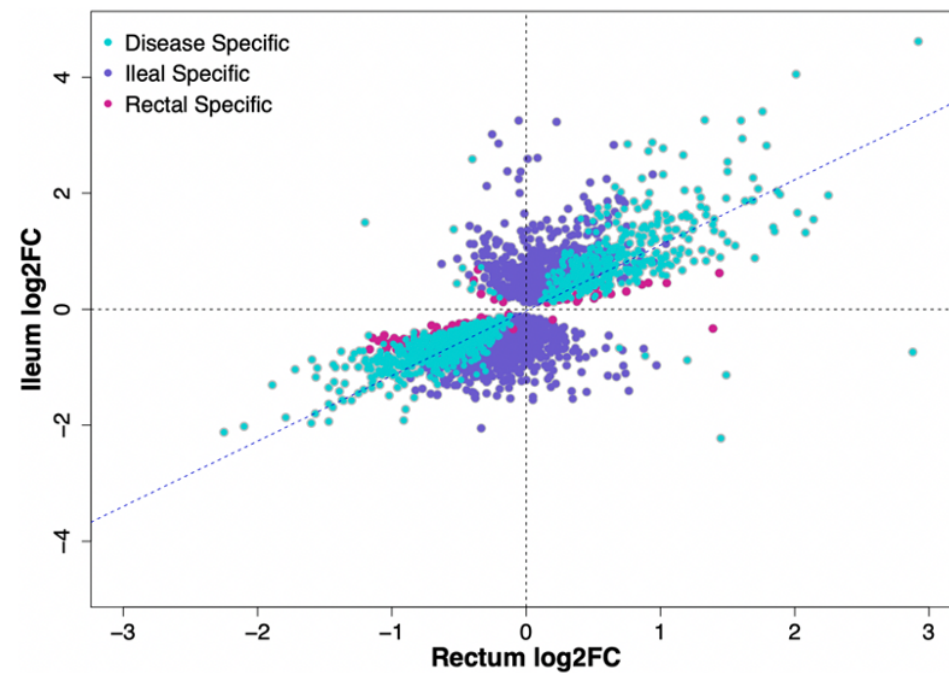

2b

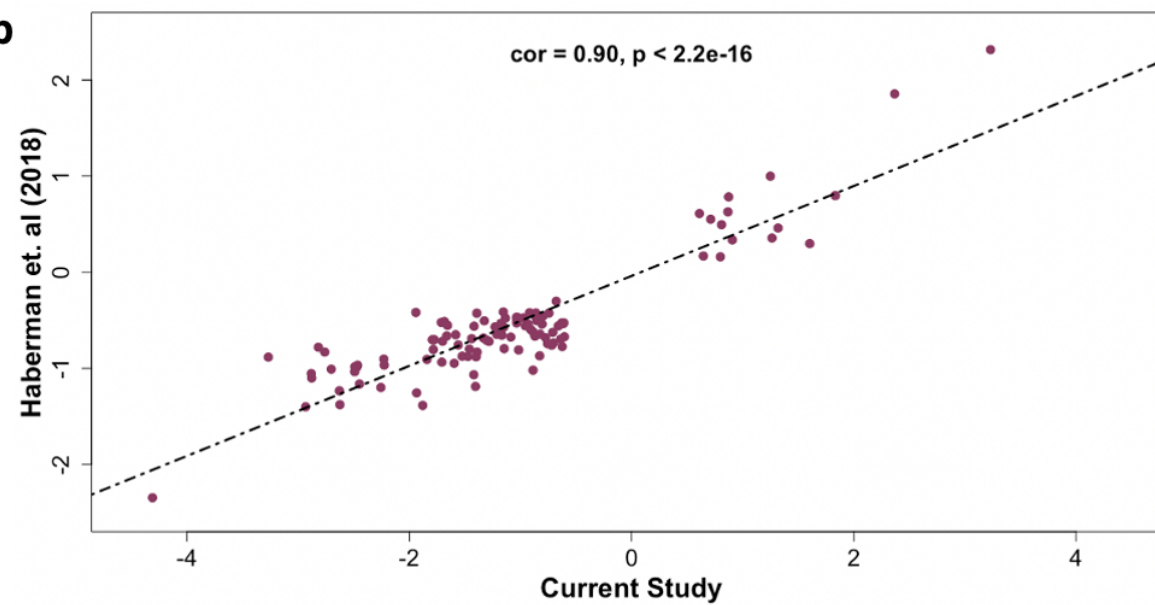

Figure S2

3a

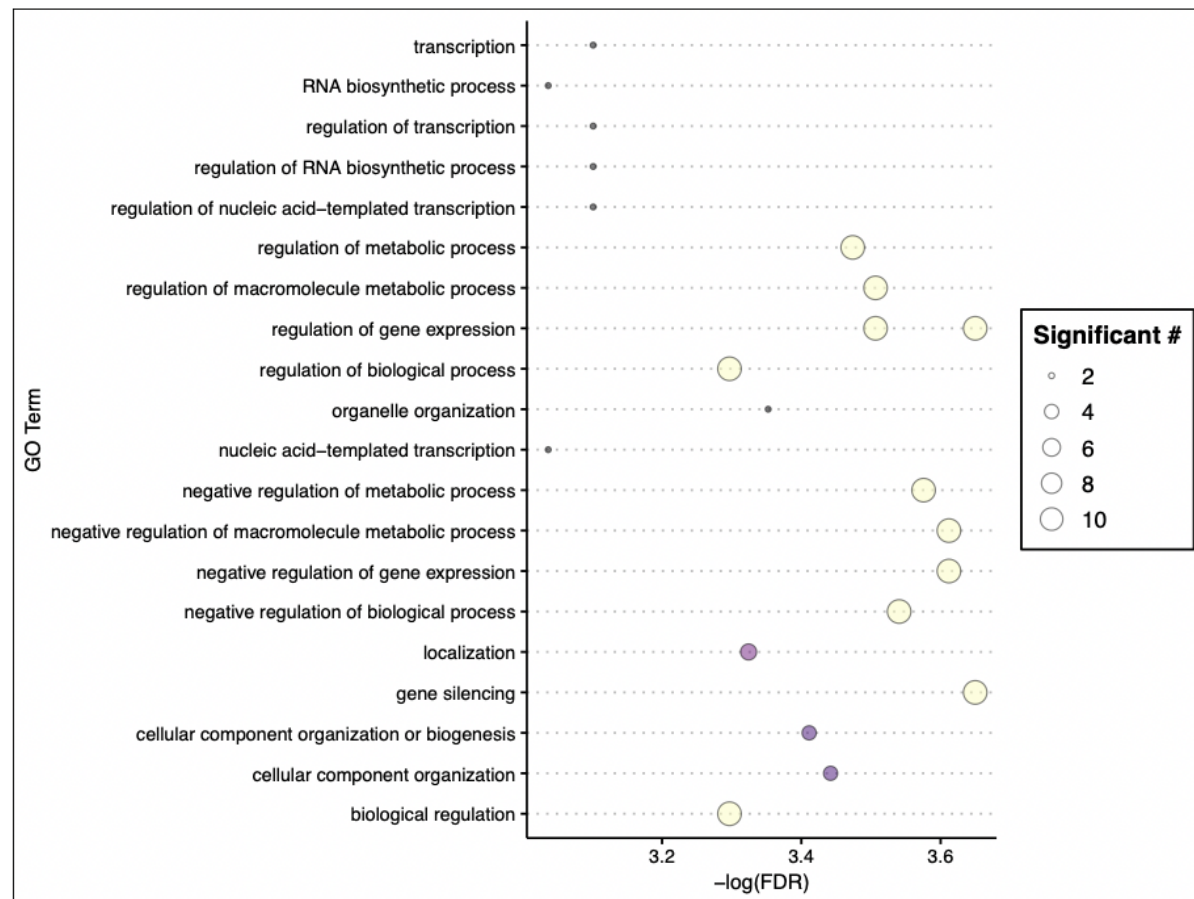

3b

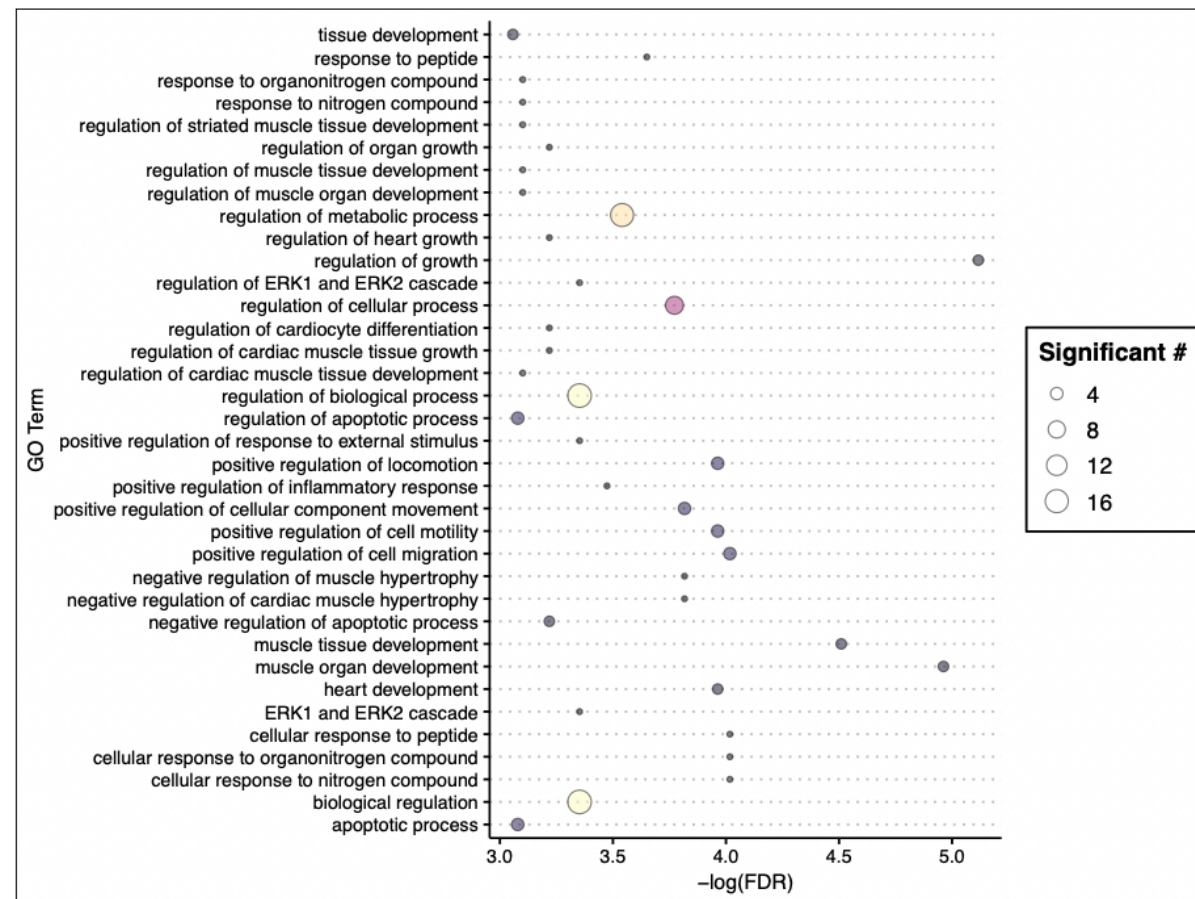

Figure S3

4a

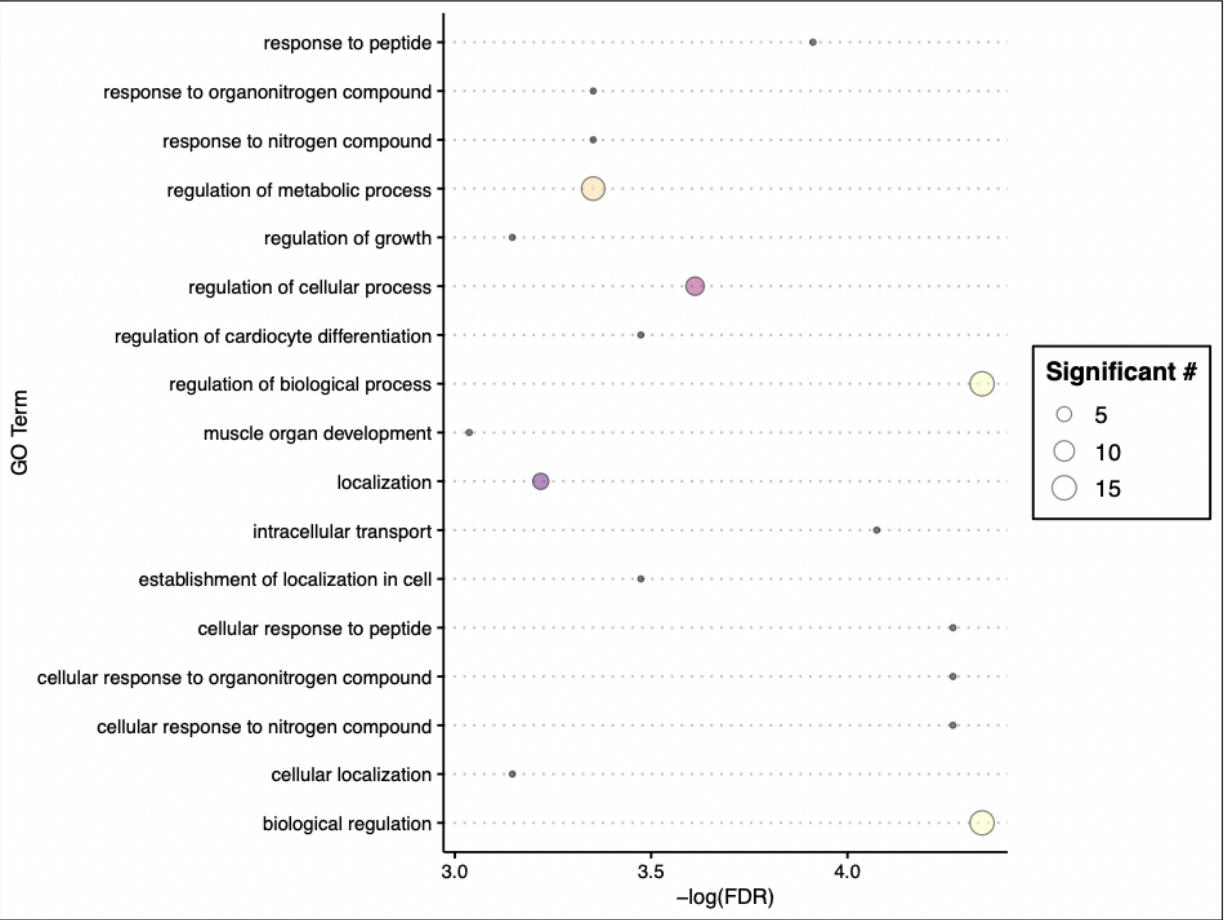

4b

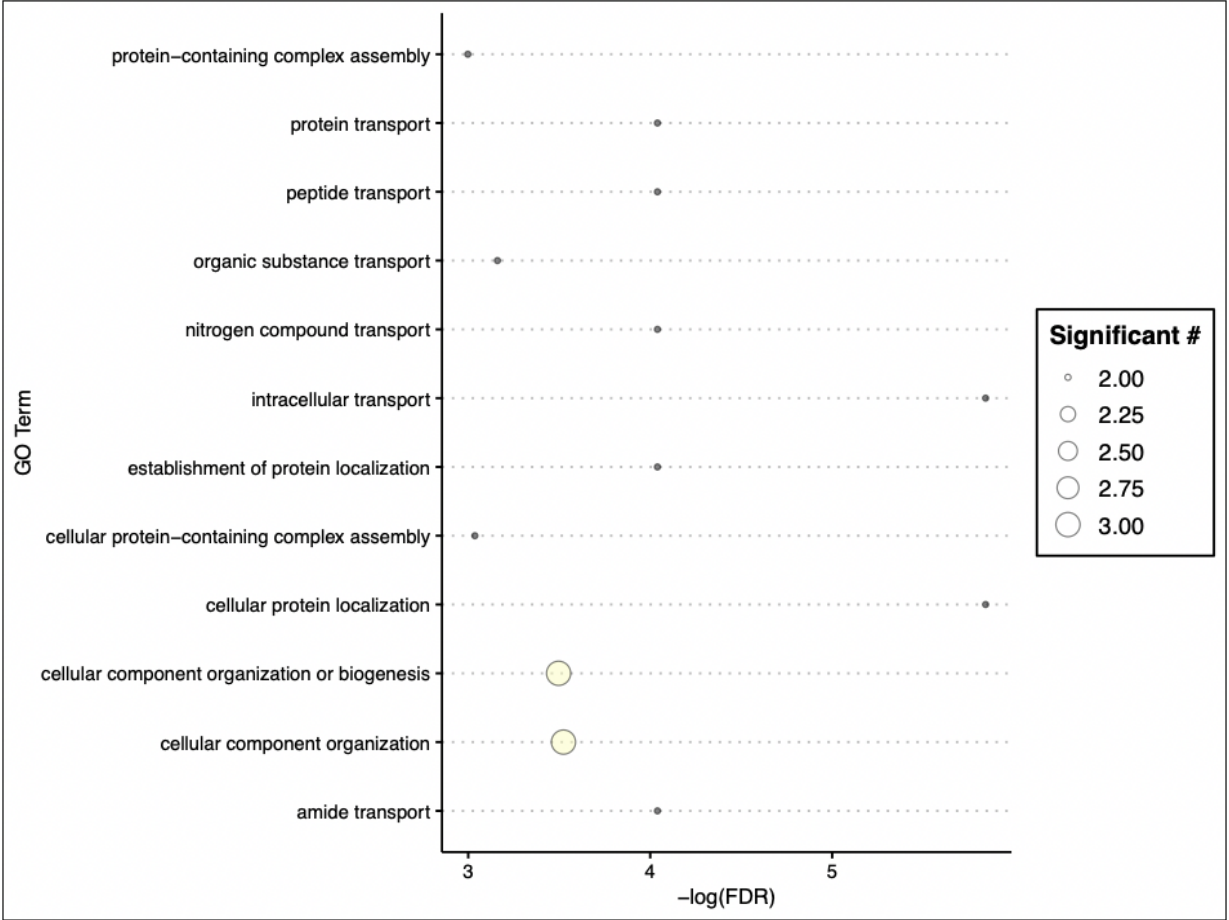

Figure S4

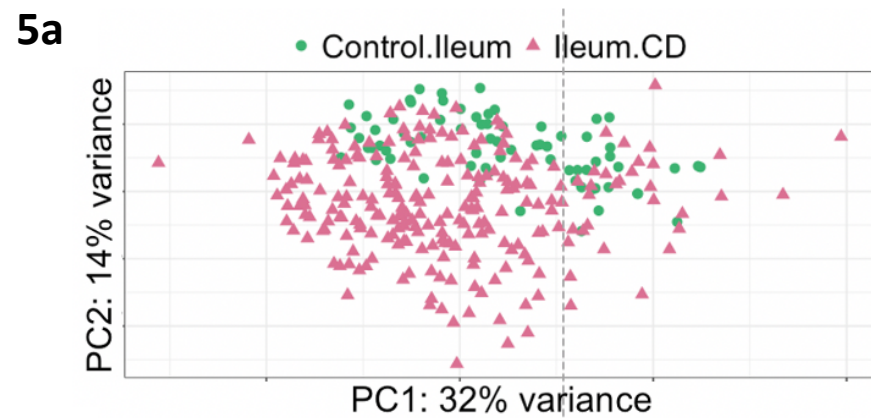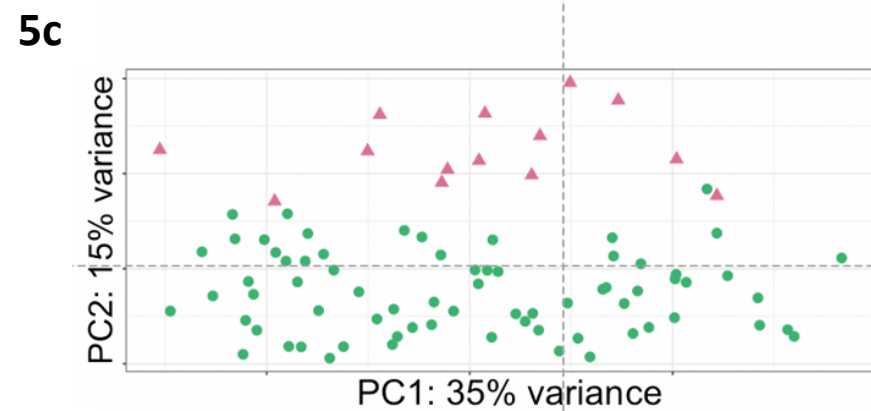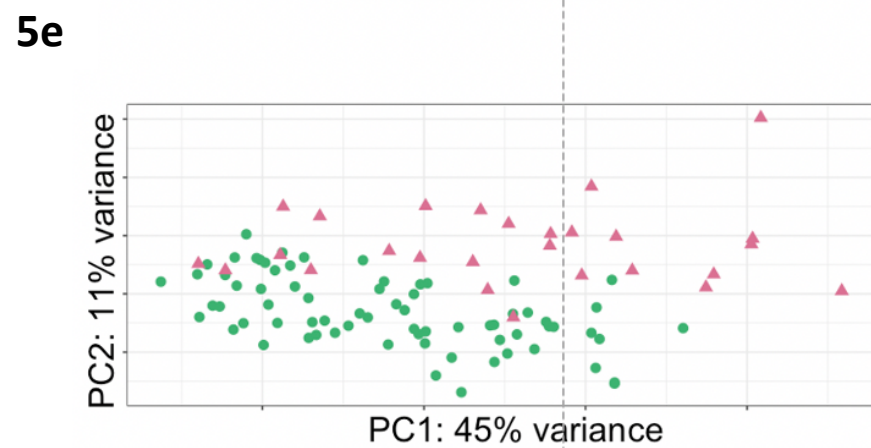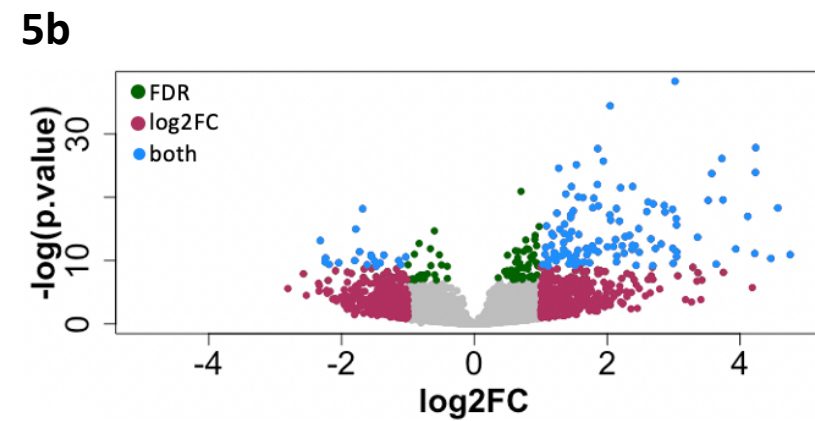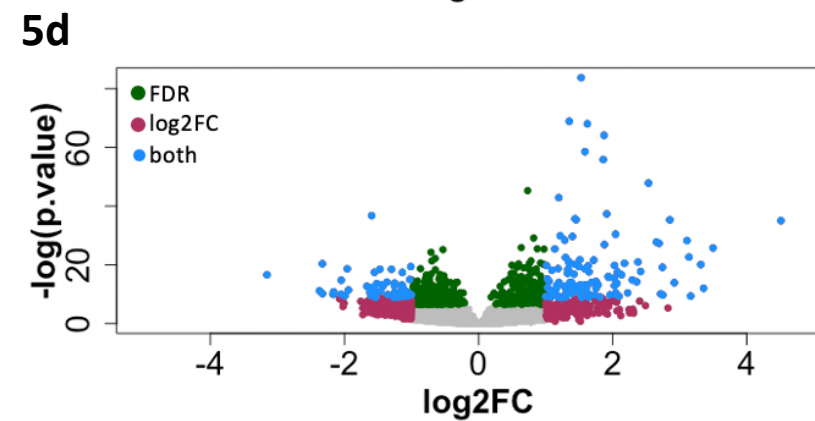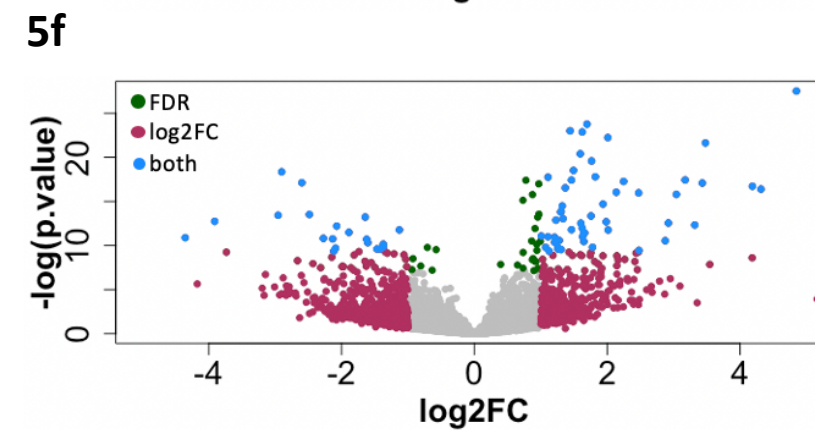

**Figure S5**

6a

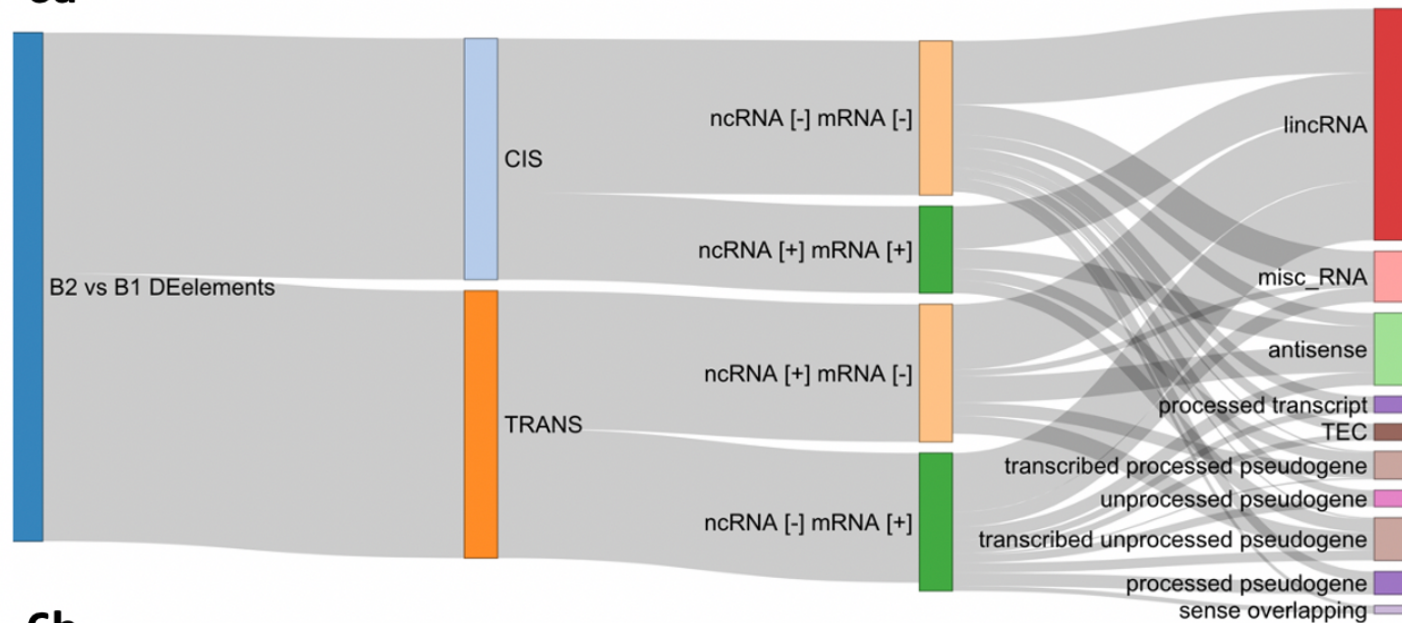

6b

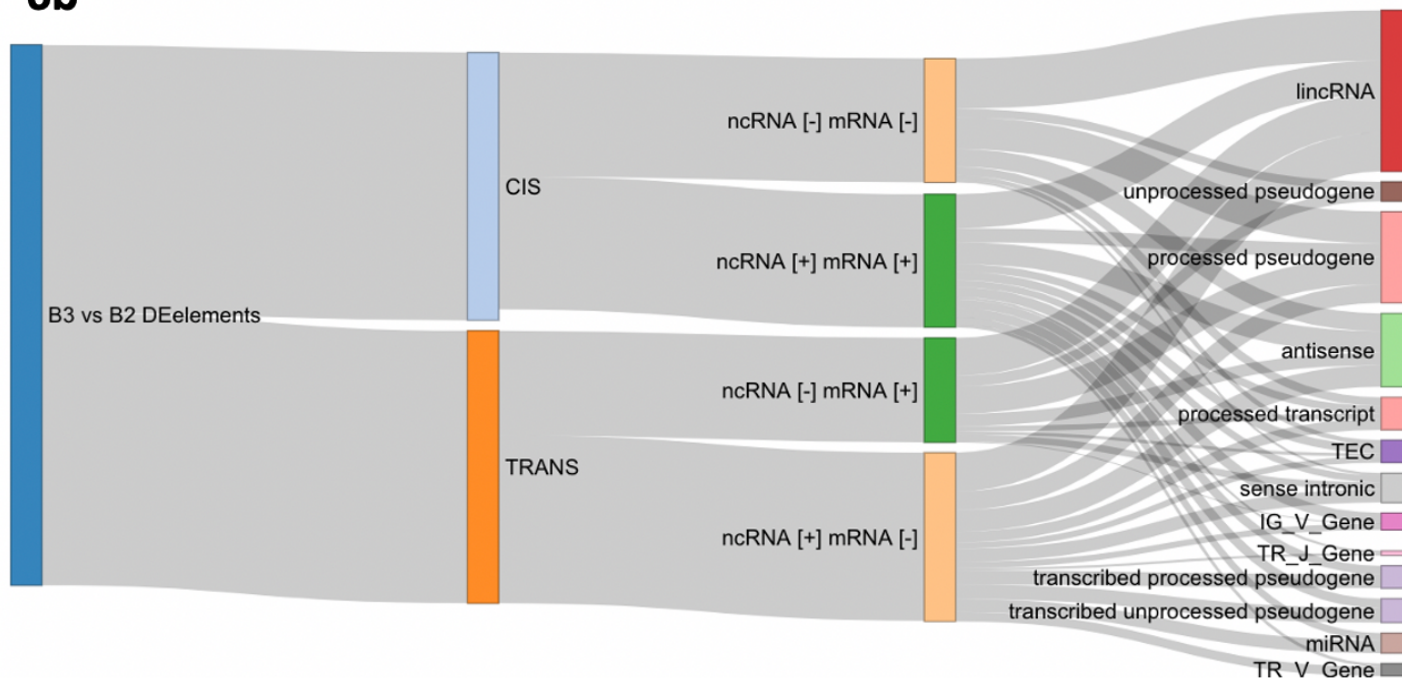

Figure S6

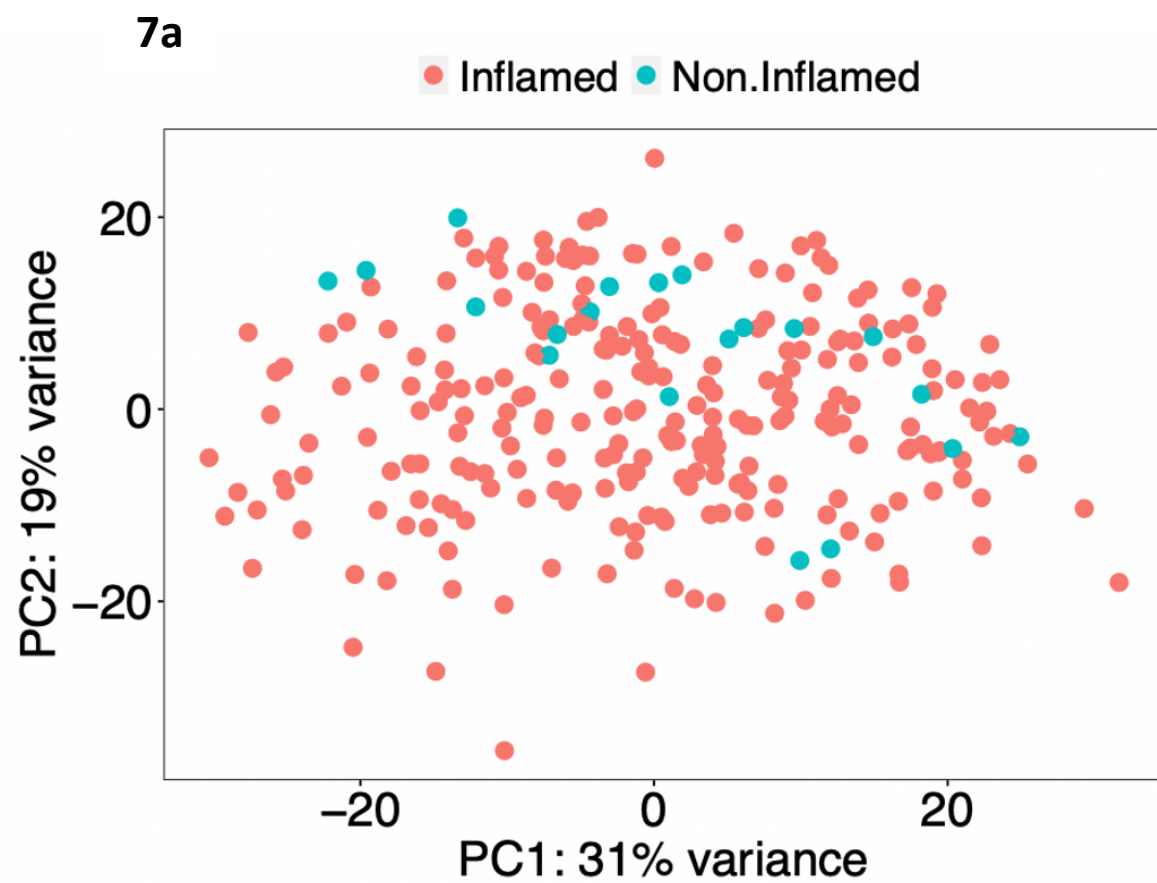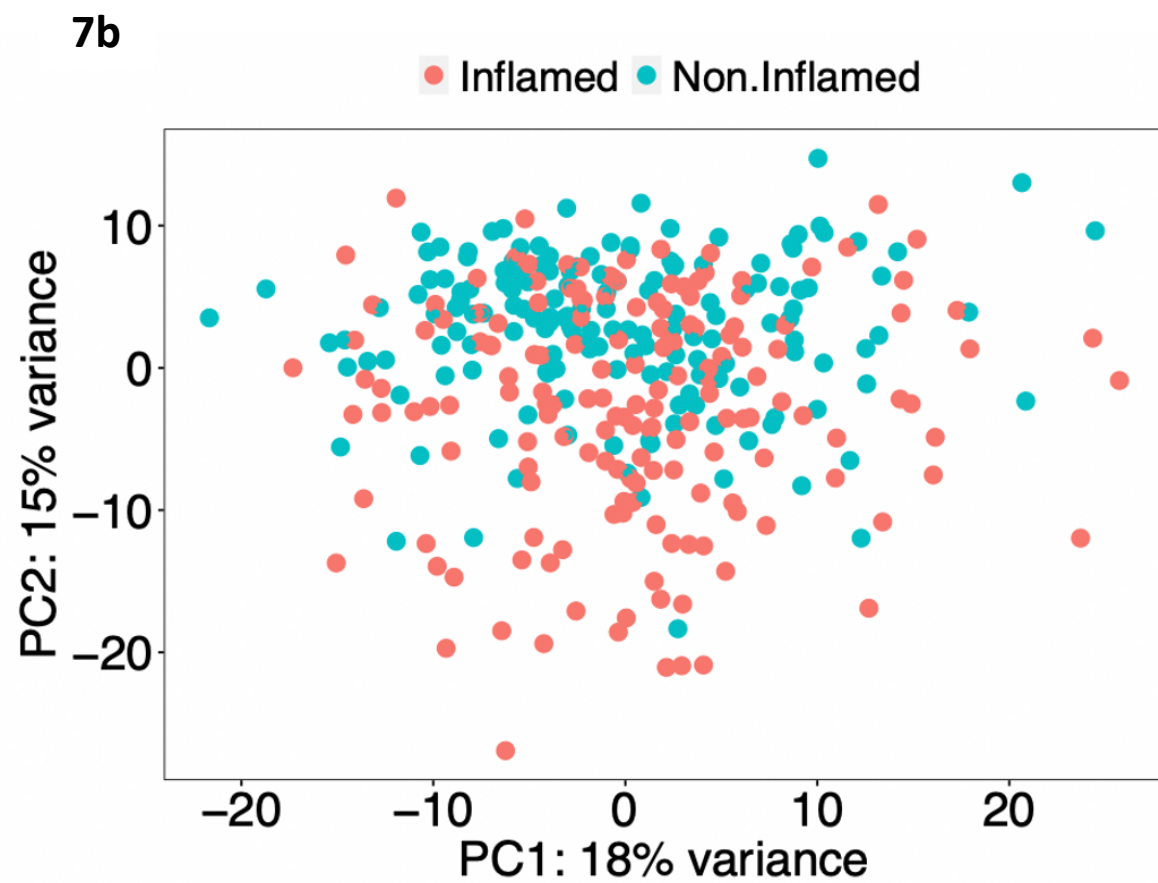

**Figure S7**

**8a**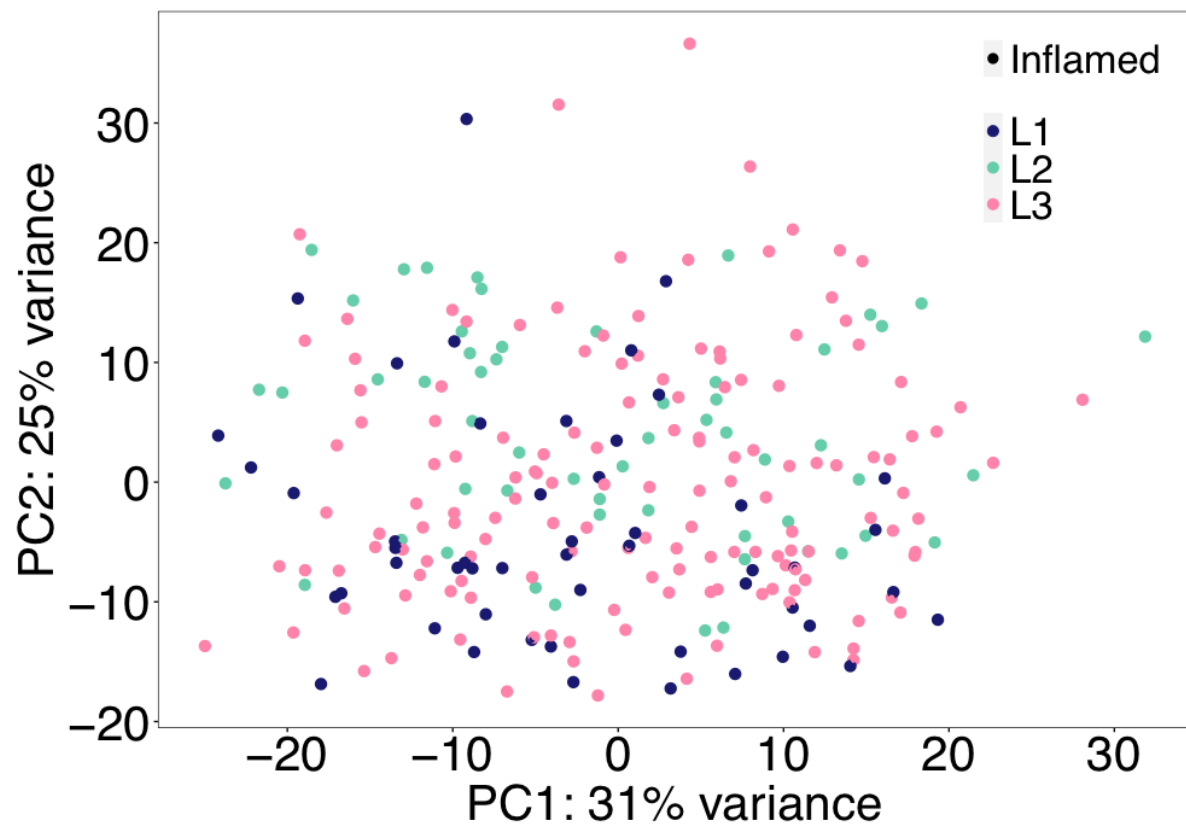**8b**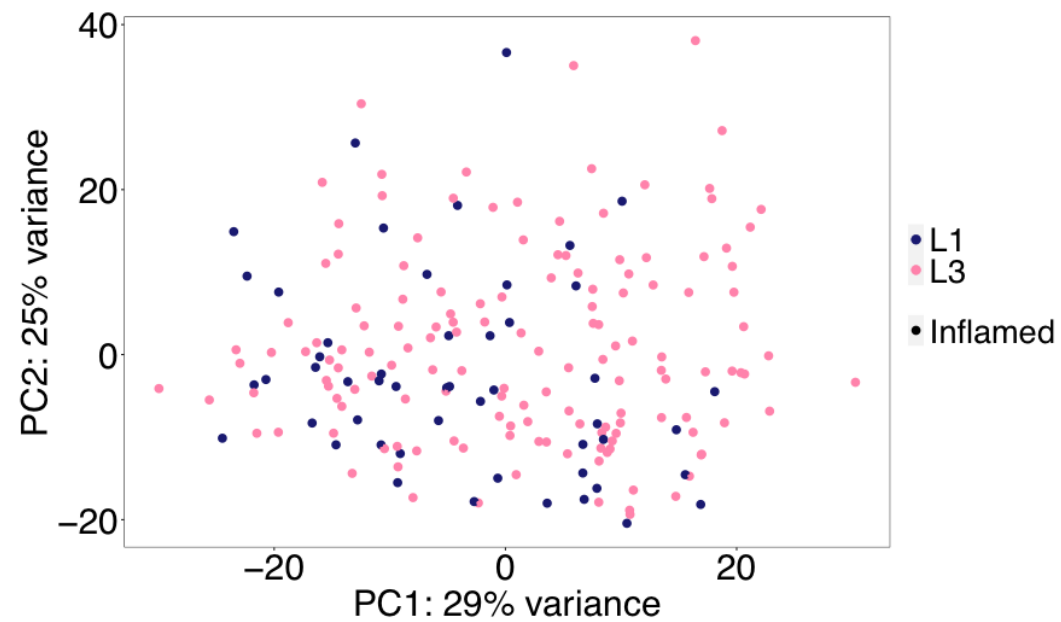**8c**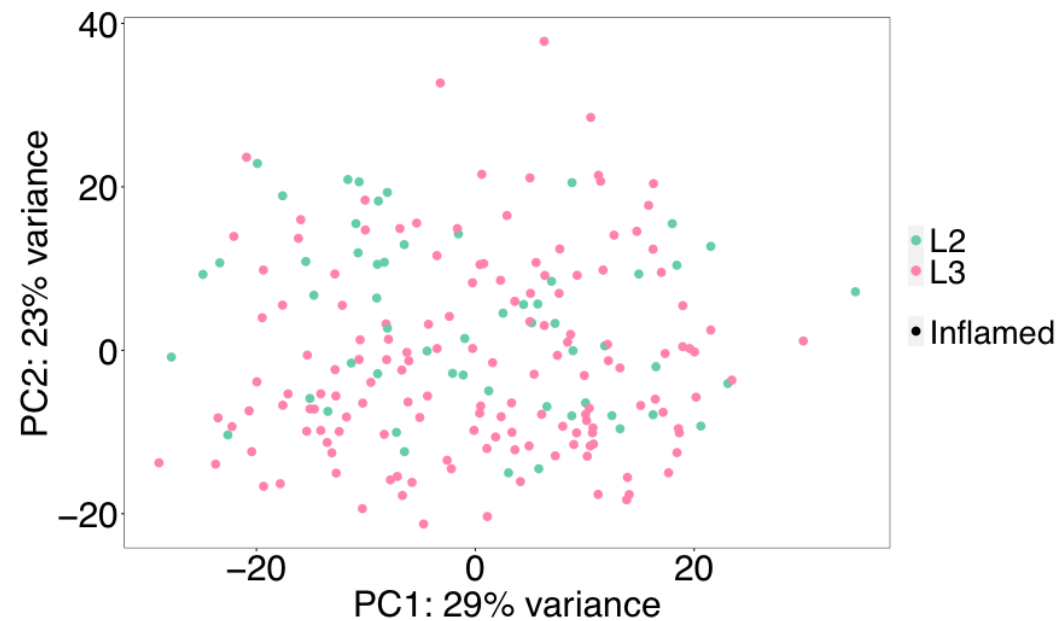**Figure S8**

Supplement: Supplementary file 2 — Additional file 2: Fig. S1. Overall Workflow of Analysis: Differential expression analysis was performed on n = 345 ileal and n = 390 rectal Bulk-RNA-later biopsies using Illumina deep-RNA sequencing in a single batch. The reference panel utilized was Hg38 with STAR package used for alignment of 58,381 transcripts, of which n = 20,779 were ncRNAs. EdgeR was used for differential expression of these ncRNAs in three comparisons: Disease Status, Disease Behavior, and Disease Inflammation. For each analysis, the total number of DE ncRNAs for ileal and rectal datasets is represented. Fig. S2. a The log2FC obtained from case-control analysis of all the 20,779 ncRNAs were compared between ileal and rectal biopsies. Each point is a ncRNA expression and they colored based on tissue- or disease-specific. b Using a subset of newly diagnosed CD patients (n = 133) from the ileal dataset (n = 345), the DE analysis results were compared with our previous study (Haberman et al. 2018) results performed on the same RISK cohort. The strong positive log2FC results shows the reliability and replicability of our analysis. Fig. S3. Gene Ontology Analysis of Crohn’s Disease versus Controls DE ncRNAs in Ileal Biopsies: Using TopGO, gene ontology analysis was conducted on n = 89 DE ncRNAs in ileal biopsies. The results displayed significant, FDR < 0.05, hits in (a) cellular components (n = 79), and b biological processes (n = 21). Fig. S4. Gene Ontology Analysis of Crohn’s Disease versus Controls DE ncRNAs in Rectal Biopsies: Using TopGO, gene ontology analysis was conducted on n = 89 DE ncRNAs in rectal biopsies. The results displayed significant, FDR < 0.05, hits in a cellular components (n = 12), and b biological processes (n = 17). Fig. S5. Principal Components and Volcano Plots of DE ncRNAs based on Crohn’s Disease Behavior: The PCs were calculated using entire list of ncRNA (n = 20,779) and first two PCs were plotted. Each point represents a subject and they are clearly separating the [file 12920_2021_1041_MOESM2_ESM.pdf]
